# Facile production of quercetin nanoparticles using 3D printed centrifugal flow reactors†

**DOI:** 10.1039/d2ra02745c

**Published:** 2022-07-19

**Authors:** Davide De Grandi, Alireza Meghdadi, Gareth LuTheryn, Dario Carugo

**Affiliations:** Department of Drug Sciences, Faculty of Pharmacy, University of Pavia Pavia 27100 Italy; Department of Pharmaceutics, School of Pharmacy, University College London London WC1N 1AX UK d.carugo@ucl.ac.uk; Department of Mechanical Engineering, Faculty of Engineering and Physical Sciences, University of Southampton Southampton SO17 1BJ UK

## Abstract

Drug nanocrystals are a delivery system comprised of an active pharmaceutical ingredient, with small amounts of a surface stabilizer. Despite offering simplicity in formulation, their manufacture can be a challenging endeavour; this is especially true when the production is performed using microfluidic devices. Although precipitation within microchannels can lead to issues such as clogging, microfluidics is an appealing manufacturing method as it provides fine control over mixing conditions. This allows production of nanoparticles with a narrower size distribution and greater reproducibility compared to batch methods. To generate microfluidic devices cost effectively, replica moulding techniques are considered the manufacturing standard. Due to its simplicity and relatively low cost, 3D printing has become prevalent at the laboratory scale, especially during iterative development of new devices. A challenge of microfluidic-based methods is that they require specialized equipment and multi-step procedures, making them less accessible to users with no previous experience. In a recent study we developed a 3D printed flow-through reactor, referred to as reactor-in-a-centrifuge (RIAC). It is a simple device designed to fit in a 50 mL tube and actuated using a laboratory centrifuge, which removes the need for specialized instrumentation. The manufacturing capabilities of the RIAC have been already proven, by reproducible production of liposomes and silver nanoparticles. The present work demonstrates the use of RIACs with a straight- and spiral-shaped channel architecture to produce quercetin nanocrystals, with therapeutically relevant size (190–302 nm) and very low size dispersity (polydispersity index, PDI < 0.1). The work focused on evaluating how changes in operational parameters (actuation speed) and formulation components (medium viscosity and stabilizer type), impacted on nanocrystal size and PDI. Under all tested conditions the obtained nanocrystals had a smaller size and narrower size distribution, when compared to those produced with alternative methods. The obtained quercetin nanosuspensions however showed limited stability, which should be addressed in future investigations. The simplicity of the RIAC makes it an appealing technology to research groups, especially in low-resource settings and without prior expertise in microfluidics.

## Abstract 2

A 3D printed reactor-in-a-centrifuge (RIAC) was developed to produce drug nanocrystals. Quercetin nanocrystals were manufactured at varying operational and formulation conditions, and had a small size (190–302 nm) and low size dispersity (PDI < 0.1).

## Graphical abstract

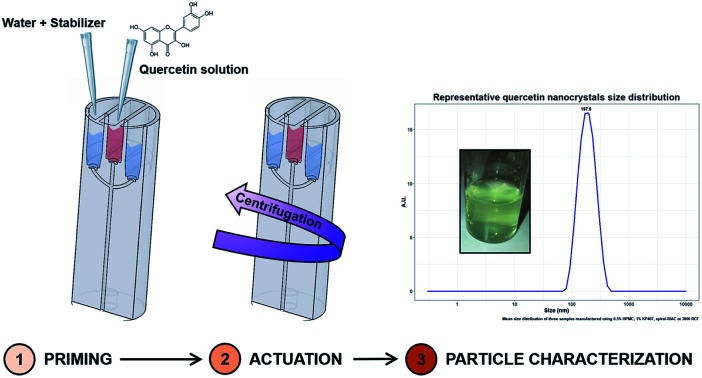

## Introduction

The nature of chemical moieties required to achieve sufficient bioactivity typically means active pharmaceutical ingredients (APIs) are highly lipophilic and hydrophobic. Evidence for this is shown by a substantial proportion of drug candidates found in class II of the biopharmaceutics classification system (BCS), where they display low solubility and high permeability; or class IV with low solubility and low permeability.^1–3^ Their physico-chemical characteristics such as reduced absorption performance and bioavailability, make these drug candidates a challenge to translate clinically.

In the last three decades, the development of nanoscale delivery systems has provided tools to address the issue of low bioavailability in many drug candidates.^4^ Depending on their properties and desired administration route, drugs have been formulated into liposomes,^5^ solid lipid nanoparticles,^6^ nano-emulsions,^7^ dendrimers,^8^ metallic^9^ or polymeric^10^ nanoparticles, and API nanocrystals.^11^ All these API delivery systems come with advantages over more classical formulations; notably, many drugs on the market will to some extent employ nanotechnology strategies to efficiently deliver an API. Some of the advantages of formulating APIs as nanoparticles are: (1) increased surface area to volume ratio, to improve dissolution rates; (2) substantially increased saturation solubility for particles smaller than 1 μm in diameter;^12–14^ (3) increased surface area favouring nanoparticle interaction with biological cells, allowing for increased adherence to mucosal layers^15–17^ and improved absorption through biological barriers;^18,19^ and (4) the nanoparticle surface can be functionalised with specific molecules to allow for increased blood circulation, greater *in vivo* stability, and organ/cell targeting.^11,20^

The terms API nanocrystals and drug nanoparticles are used interchangeably, to describe a specific type of drug delivery system. They are composed of the drug itself in the form of extremely fine solid particles, with the addition of small amounts of surface coating stabilizers to minimise inter-particle aggregation.^21–23^ API nanocrystals can be manufactured by top-down, bottom-up or combination methods. Top-down techniques aim to reduce the particle size of coarse drug powder down to the nanometric scale; they are based on high energy methods such as wet media milling or high-pressure homogenization (HPH) that rely on shear and impact forces.^24^ Bottom-up techniques allow nanoparticle production through controlled precipitation, starting from an API solution. The most used bottom-up techniques are solvent evaporation and solvent-antisolvent precipitation; both of these methods exist in several variations and allow for rapid and controlled onset of the supersaturation state, which initiates crystal nucleation, growth and subsequent nanoparticle precipitation.^25^ Combination techniques instead involve multiple size reduction processes that usually include a first micro or nanoprecipitation step followed by HPH, but many variations of this technique are described in the literature.^26–28^

Currently, the vast majority of marketed nanocrystal formulations are manufactured using top-down approaches.^29^ However, promising alternative manufacturing technologies have been developed in the last decades. One approach is microfluidic solvent-antisolvent precipitation.^30,31^ In this method supersaturation is achieved by the addition of an antisolvent (usually water) to an organic solution of the API, which causes nucleation and precipitation of drug particles.^25,32^ The precipitation process comprises three stages, as postulated by the LaMer mechanism.^33^ Briefly, the first stage is characterized by an increase in solute concentration or a reduction of its solubility, to reach the minimum solute concentration needed for inducing nucleation. This is followed by a rapid nucleation process that leads to a significant reduction in solute concentration, which consequently reduces the nucleation rate to zero. The last stage is characterized by the growth of all nuclei caused by deposition of solute molecules on their surface. The growth process allows nuclei to become large enough to exist as solid particles, in equilibrium with the surrounding suspension. When the mixing of solvent and antisolvent is rapid enough to determine a homogeneous supersaturation state in the whole solution, nucleation and growth processes occur evenly throughout a sample; leading to the production of particles with low size dispersity.^34^

The purpose of a microfluidic device in this process is to enable rapid and highly reproducible mixing between solvent and antisolvent, ensuring that the final product is of optimal standard. Numerous systems exploiting this technology have been developed in recent years.^31,35–39^ Considering differences between these systems, microfluidic devices for nanoprecipitation share common advantages over batch methods. The confined space of microchannels allows the manipulation of small liquid volumes, which reduces the mixing path of solvent and antisolvent. This in turn decreases the time needed to achieve complete mixing (referred to as mixing time), which is a key parameter governing nucleation and crystal growth. Moreover, the flow dynamics within microchannels is easier to predict and characterize than in batch methods, ensuring reproducible mixing conditions. Finally, if scale-up remains a challenge (particularly for pre-clinical research purposes), microfluidic chips can be operated in parallel and under continuous-flow regime, allowing for increased production rates.^32,35,37–40^

Despite more than two decades of continued research, the utility of microfluidic devices is not without limitations. Productivity at industrial scales is possible *via* device parallelization^39,41^ or the design of chips capable of withstanding greater flow rates,^30,39^ but it requires significant development and optimization time. Additionally, most microfluidic devices require expensive syringe or pressure-controlled pumps for their operation, which hinders translation and widespread adoption of these techniques.^31^ Furthermore, channel clogging caused by the accumulation of precipitated nanoparticles remains an issue,^32,36,42^ and fluid leakages can occur at connections between the microfluidic device and pump units as a result of this clogging or inefficient sealing. Despite the challenges involved, recent studies have highlighted the research progress made to develop microfluidic devices that can sustain greater flow rates (including patented designs).^39^

Previous research has explored alternative microfluidic device designs that are operated without the use of expensive pumps.^43–45^ However, similar challenges related to cost, scalability and device lifetime remain a concern if these approaches were to be adapted for the synthesis of nanoparticulate systems. In a recent study we have reported on the development of a 3D printed flow-through reactor, referred to as reactor-in-a-centrifuge (or RIAC).^46^ The RIAC is a single-piece fluidic device embedded in a cylindrical body, which is designed to fit into a standard 50 mL centrifuge tube. The RIAC features reservoirs for storing reagents, which then connect through a junction to form a mixing channel. The force required to drive fluids through the mixing channel is provided by a laboratory centrifuge. We have previously demonstrated that this system can be employed to manufacture silver nanoparticles and liposomes, with dimensional properties that are comparable or superior to those of batch and conventional microfluidic production methods.^46^

The RIAC concept sought to overcome some of the key limitations of more conventional microfluidic-based devices. Notably, it can be operated with any centrifuge model that can host a 50 mL Falcon tube, which is a piece of equipment most labs are likely to find accessible. Since there is no need for hydraulic pumps, the risk of leakages is practically absent. The simplicity of operation allows users without any prior expertise in microfluidics to quickly integrate this method in their research. Moreover, the 3D printed RIAC can be used immediately after manufacturing, whereas silicone-based microfluidic devices obtained *via* soft lithography typically require both long curing times and are prone to failure due to ineffective sealing. Lastly, increasing the scale of the channel diameter from tens or hundreds of micrometres to more than one millimetre, aids in prevention of channel clogging and offers a means of increasing the scale of production per-device. However, the ease of use and convenience of the RIAC in formulation development come at some costs. As the device is operated within a closed centrifuge, it is very complex to integrate on-line quality control systems that would also provide insights into the nanoparticle production process. For this reason, the RIAC is less suitable than other reported techniques for studying nanoparticle formation mechanisms or observing the transport of fluids and chemical species within microchannels.^47–51^

Building upon our previous study, in the present work we aimed to determine whether the RIAC can be employed as a tool for rapid, reliable and facile production of API nanocrystals. In particular, we report on the design and manufacturing of two different RIAC prototypes, the development of a method for API nanoparticle production using these devices, and a comparison of nanoparticles obtained through this method with those reported in the literature. The study also aims to contribute to the development of production methods for drug delivery systems that have potential for rapid widespread adoption, including within low-resource settings.

The model drug chosen for this study was quercetin[2-(3,4-dihydroxyphenyl)-3,5,7-trihydroxychromen-4-one]; a secondary metabolite present in a wide variety of plants that belongs to the flavanol subclass of flavonoid compounds. Quercetin is a useful model drug in this context for a number of reasons. Firstly, it is a natural compound with a spectrum of bioactive properties, which range from anti-oxidant, anti-inflammatory, and anti-tumoral to anti-bacterial and anti-viral.^52–55^ Many studies have already demonstrated its efficacy *in vitro* and *in vivo*, but to the best of the authors' knowledge there is no marketed pharmaceutical product that utilises quercetin as its principal active component. Secondly, quercetin can be obtained in the form of nanocrystals both *via* bottom-up and top-down methods as reported in the literature, which allows a comparison between the RIAC and alternative production methods to be made.^56–59^ Lastly, quercetin is a naturally fluorescent molecule. This innate feature is advantageous for multiple stages of research, as it facilitates visualization of nanocrystals in complex matrices or formulations, as well as in biological assays (*in vitro* or *in vivo*) using fluorescence imaging techniques. Imaging of these particles therefore would not require labelling with an additional fluorescent molecule that could alter their physico-chemical properties.

To the best of the authors' knowledge, this is the first study reporting on the manufacturing of drug nanoparticles using a centrifuge-actuated 3D printed flow-through reactor. To facilitate replication and adoption of the device by other laboratories, technical drawings of the RIAC (in .stl format) are also provided as ESI† to this manuscript.

## Materials and methods

### Materials

Quercetin (>95% HPLC grade), hydroxypropyl methyl cellulose (HPMC), Kolliphor P 407 (KP407), Kolliphor P 188 (KP188), Tween 20 (TW20) and absolute ethanol (99.8%) were all purchased from Merck Life Science UK Limited. HPMC has a viscosity of ∼15 mPa × s as a 2% solution in DI water, measured at 25 °C. Purified water was supplied through the Purite Select D80 GP purification system. Tough polylactic acid (tough PLA) was employed to 3D print the RIACs and was purchased from Ultimaker BV.

### Design rationale of RIACs

**Fig. 1:**
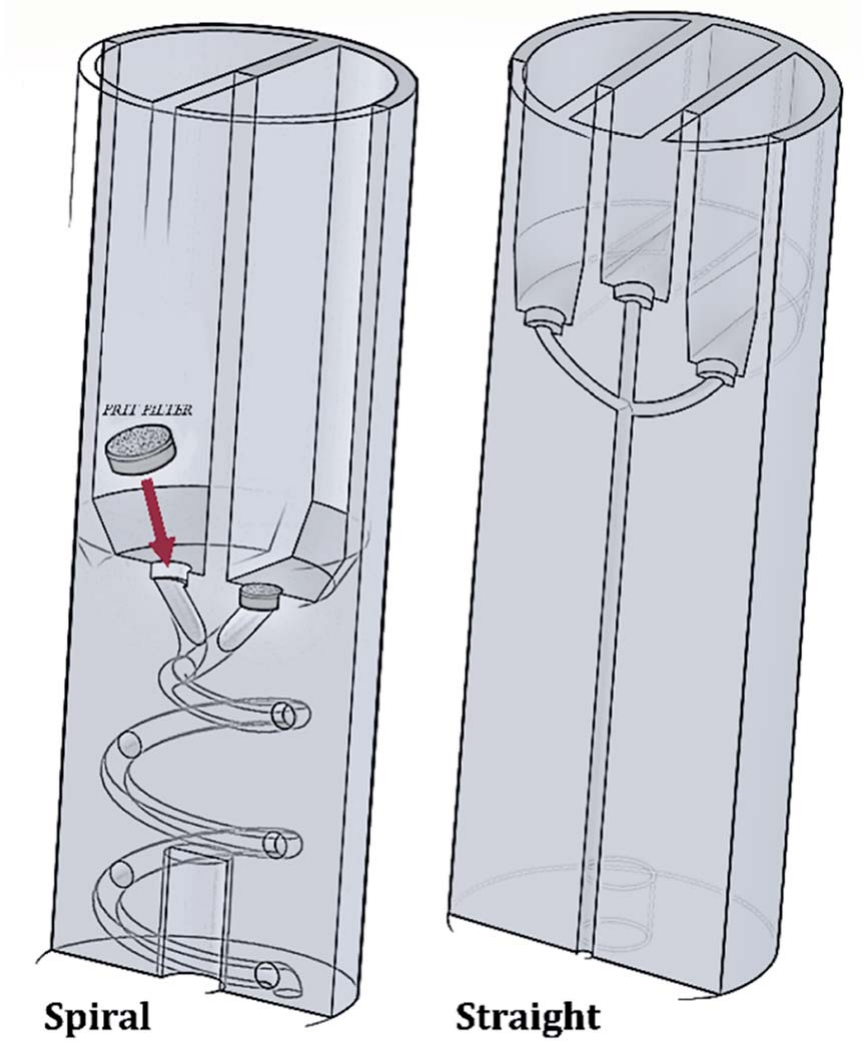
Schematic representation (cross-sectional view) of both spiral- and straight-RIAC prototypes. Both RIACs contain a recess at the bottom of each reservoir, to host a 3.175 mm steel HPLC-grade FRIT filter. The positioning of these filters is illustrated for the spiral-RIAC.

**Fig. 2:**
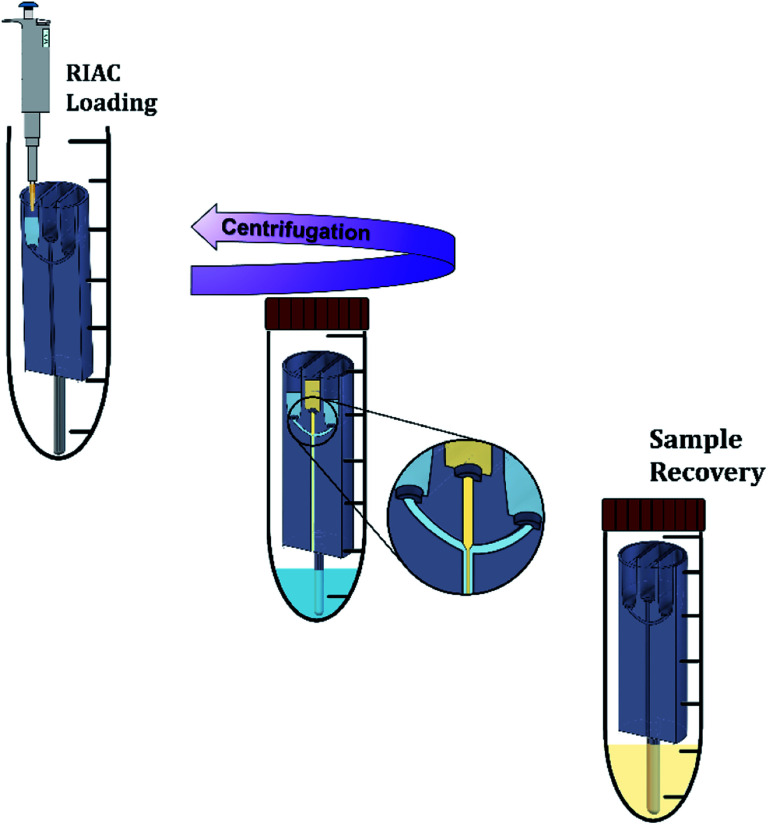
Nanocrystals production method using the RIAC. The steel support is connected to the RIAC, and the reactor is placed in a 50 mL centrifuge tube. Using a micropipette, reagents are added to the reservoirs and to the bottom of the tube. The tube is then closed and placed in the centrifuge. In this work, two reactors were actuated simultaneously in each run. After centrifugation, the reactors are removed from the tube, and the sample is recovered in a glass vial. The reactors are then washed with absolute ethanol and dried as described in the Methods section.

The RIAC was designed using the computer-aided drawing (CAD) software (Autodesk Inventor®), and subsequently 3D printed from Ultimaker proprietary ‘tough’ PLA using a fused deposition modelling (FDM) printer (Ultimaker S5). Two different RIAC configurations were employed in this study, referred to as spiral- and straight-RIAC respectively (Fig. 1). The spiral-RIAC is the same device employed in our previous study, and is characterized by a spiral shaped mixing channel.^46^ The straight-RIAC is instead a novel design that comprises three reservoirs and a straight mixing channel, which is a configuration comparable to the one used in hydrodynamic flow focusing microfluidic devices. Both reactors consist of four main features: the reservoirs, the inlet channels that originate from the reservoirs, a junction between inlet channels, and a mixing channel. All these features are embedded within a cylindrical body, which is designed to fit within a standard 50 mL centrifuge tube (Fig. 2).

The spiral-RIAC contains two identical reservoirs connected to the mixing channel through a Y-shaped junction between two inlet channels. The mixing channel has a radius of 1 mm, radius of curvature of 6.5 mm, length (excluding the junction) of 102 mm, and displays a total of 2 revolutions. Overall, the device is 24 mm in diameter and 70 mm high. The straight-RIAC instead contains three reservoirs. As shown in Fig. 1, the lateral reservoirs were designed to be higher than the central one, to ensure that the length of the central inlet channel was the same as for the lateral ones. The mixing channel has a radius of 1.25 mm and length (excluding the junction) of 44.8 mm. The device has the same overall diameter and length as for the spiral-RIAC.

It should be noted that the two RIAC configurations represent two alternative prototypes and that the effect of a single specific design feature cannot be inferred from the present investigation, since different design characteristics were simultaneously varied to optimise each specific RIAC configuration. For example, reservoirs in the straight-RIAC had reduced cross-section and height in order to maximise the mixing channel length, and in turn increase mixing efficiency between solvent and antisolvent. Both RIACs were however run with the same liquid volumes, to facilitate comparison of performance between the two devices.

A challenge with the proposed centrifugal reactor concept is to prevent reagents from flowing downwards through the mixing channel before actuation. For this purpose, HPLC-grade FRIT filters (pore size: 0.5 μm) were placed within a recess that was fabricated at the bottom of each reservoir, as described previously^46^ (see Fig. 1). Given the small pore size of these filters, they are capable of effectively retaining fluids within the reservoirs upon priming. Laminar flow through the pores is also expected once the device is actuated.^60^ In addition, the filters potentially allow for the production of ‘cleaner’ end-products, as they can prevent dust particles and small precipitates from flowing into the mixing channel.

Both RIAC prototypes were designed to avoid printing of support material. The fabricated single-body cylinder is therefore ready-to-use right after manufacturing, without requiring any post-production step. In our previously reported design,^46^ a 3D printed bottom-support was also manufactured and coupled with the cylindrical body to provide space at the bottom of the Falcon tube where the reaction products would collect. In this study, it was found that the support underwent progressive deformation when the RIAC was operated at higher relative centrifugal forces (>1800 RCF), which became apparent after about 10–15 consecutive runs. The 3D printed support was thus replaced by a stainless-steel rod (diameter: 5.2 mm, height: 4.8 cm). With this modification, the device maintained its physical integrity and overall performance even after many cycles of operation (>50 at the point of writing).

### 3D printer settings for RIAC manufacturing

The Ultimaker CURA software (Version 4.12.1) was employed to define the 3D printing process settings. The layer height was set to the lowest possible value (0.06 mm) to obtain a smooth surface finish within channels. Other parameters were defined as follows: bottom/top thickness = 1.4 mm, infill density = 60%, infill pattern = grid, printing speed = 25 mm s^−1^, and nozzle size = 0.4 mm. Using these settings, only 25 g and 21 g of tough PLA were required to manufacture the straight-RIAC and spiral-RIAC, respectively. Although a lower PLA consumption could have been potentially achieved, it was decided to manufacture a device that was mechanically robust enough to withstand many operation cycles.

During the printing process, reservoirs were oriented upward to avoid the creation of support material within them. This also allowed for accurate manufacturing of the reservoirs' bottom surfaces and frit seats. Overall, both RIAC configurations could be printed without the need for support material, as detailed in our previous study.^46^

### Protocol of quercetin nanocrystals production

For nanocrystals production, quercetin was solubilized in absolute ethanol at a concentration of 9 mg mL^−1^. The saturation solubility of quercetin in ethanol is of approximately 12–15 mg mL^−1^ at 25 °C. Quercetin concentration in the experiments was thus kept below the solubility limit to reduce the risk of precipitation, which could be due to room temperature being <25 °C or caused by ethanol evaporation from the container. After complete solubilization, the ethanolic solution was filtered through a 0.20 μm pore size Millex®-GN syringe filter (Merck Millipore Ltd, UK). All polymers (Kolliphor P 407, Kolliphor P 188, Polysorbate 20 and hydroxypropyl methyl cellulose) were solubilized in purified water at different concentrations (between 1% and 4% w/v). After complete solubilization, the solution was also filtered through a 0.20 μm pore size Millex®-GN syringe filter (Merck Millipore Ltd, UK). Solutions were prepared in batches (80 mL), so that a single batch could be used to manufacture every sample in triplicate. A single quercetin solution batch (60 mL) was used to manufacture every sample. All solutions were prepared, stored and utilised for nanocrystals production at room temperature (∼21 °C). Quercetin and polymers concentrations were chosen on the basis of previous studies reporting on the production of quercetin nanocrystals.^11,32,56–59^

The nanocrystals production method is illustrated in Fig. 2. Initially, the steel bar support was mounted and the empty RIAC was placed within the centrifuge tube. Liquid media were then added to the reservoirs. In particular for the straight-RIAC, 0.5 mL of the polymer aqueous solution (antisolvent) were pipetted in the two lateral reservoirs. Subsequently, 0.5 mL of the quercetin ethanolic solution (solvent) were pipetted in the central reservoir. Lastly, 3.5 mL of the polymer aqueous solution were placed at the bottom of the centrifuge tube (Sarstedt® polypropylene 50 mL, 114 × 28 mm, conical bottom). This additional amount of liquid added at the bottom of the tube reduces the final ethanolic concentration (preventing nanocrystals solubilization) and may discourage interparticle aggregation. In previous work, this approach has proven beneficial in reducing particle size and size dispersity of liposomes produced using the RIAC.^46^ For the spiral-RIAC, 0.5 mL of polymer aqueous solution were pipetted in one of the two reservoirs, whilst 0.5 mL of quercetin ethanolic solution were pipetted in the other reservoir. Lastly, 4 mL of polymer aqueous solution were added at the bottom of the centrifuge tube. The tube was then closed and placed inside a centrifuge rotor. A swing-out rotor was employed in this study, although liposomes were successfully produced by RIAC using a fixed angle rotor in previous work.^46^ It was hypothesised that a swing-out rotor could be more effective and reproducible at driving fluids through the RIAC, as the centrifugal force would act perpendicularly to the cross-section of the reservoirs. In this work, the centrifugation time and relative centrifugal force (RCF) applied were optimized in preliminary tests. Upon optimization, RIACs were operated either at 3000 RCF for 3 minutes or at 500 RCF for 8 minutes.

Each RIAC produced 5 mL of particle suspension containing 4.5 mg of quercetin per centrifuge run, for both reactor configurations. After each run the RIAC underwent a three-step cleaning process, by being washed with absolute ethanol before adding 1 mL of absolute ethanol in each reservoir and running the RIAC at 3000 RCF for 3 minutes. The RIAC and centrifuge tubes used were then washed again with absolute ethanol and air dried.

This work investigated the effect of varying the RCF, the type of particle stabilizer, the concentration of viscosity enhancer (HPMC) and the RIAC configuration, on the dimensional properties of the obtained nanoparticles. All experiments were conducted at room temperature (∼21 °C), which is consistent with previous studies reporting on microfluidic-based production of drug nanocrystals.^32,61–63^

### Characterization of quercetin nanocrystals

Dynamic light scattering (DLS) measurements were performed to determine quercetin nanocrystal mean diameter and size dispersity, using the Zetasizer Ultra instrument (Malvern Instrument Ltd, UK). For each measurement, 20 μL of suspension were diluted with 980 μL of deionised (DI) water, which were previously filtered using a 0.20 μm syringe filter. For DLS measurements, 1 mL disposable Fisherbrand™ polystyrene cuvettes (Fisher Scientific Ltd, UK) were used. Each sample underwent an equilibration time of 120 s and measurements were carried out at 25 °C. Three measurement runs were carried out per sample, where each run comprised 5–7 scans. Preliminary samples containing coarse precipitated nanocrystals, with diameter < 10 μm, were only measured once and 12–18 scans were carried out to confirm that the particle size was too large for further analyses. The DLS analysis settings were defined as follows: material refractive index = 1.77, material absorption coefficient = 0.4, solvent refractive index = 1.33, and solvent viscosity = 0.8872 mPa × s. The average nanocrystal size (expressed in terms of peak mean of the size distribution) and the peak width were obtained from the Zetasizer software (ZS Xplorer, Version 2.2.0.147). The polydispersity index (PDI) was employed as a measure of nanocrystal size dispersity and calculated as (peak width)^2^/(peak mean diameter)^2^.

Zeta potential measurements were also performed using the Zetasizer Ultra instrument (Malvern Instrument Ltd, UK). For each measurement, 50 μL of suspension was pipetted at the bottom of a DTS1070 Fisherbrand™ polystyrene folded capillary cell and diluted with enough DI water (filtered through a 0.20 μm syringe filter) to cover half of the electrodes (∼900 μL). Three measurement runs per sample were carried out, each comprising 10–100 scans. The default voltage level of 150 V was employed. The parameter values for the analysis were set as follows: material refractive index = 1.77, material absorption coefficient = 0.4, solvent refractive index = 1.33, solvent viscosity = 0.8872 mPa × s, and solvent dielectric constant = 78.5 F m^−1^. Results were expressed as mean zeta potential and zeta potential distribution.

### Centrifugal settings for RIAC actuation

The RIAC was operated using a Sigma 3–16 KL refrigerated centrifuge with a Sigma 11 180 swing-out rotor, which comprised four 13 190 round buckets and four 17 344 tube holders. RIACs were operated between 200 and 4000 RCF, as it was established that within this range reservoirs emptied fully and consistently across repeats. When RCF values < 200 were employed, emptying was often incomplete and less consistent across repeats. The centrifugation time was set depending on the RCF value applied (2–8 minutes as specified below in the preliminary tests section).

### Sample post-processing to improve suspension stability

Upon production it was observed that the quercetin nanocrystal suspension precipitated irreversibly in the form of large aggregates after 4 to 12 hours. It was therefore decided to evaluate two post-processing methods to enhance nanocrystal surface coating by the polymeric stabilizers. Both these methods were applied just after sample production. One approach involved stirring the sample for 30 min at 800 rpm, using the Fisherbrand Isotemp Hot Plate Magnetic Stirrer 7′′ × 7′′. In the other approach, the sample was processed using the IKA® Ultra-Turrax T 18 basic homogenizer, mounting the S 18 N–10 G dispersing tool, at speed level of 4 (∼15 600 rpm) for 10 minutes.

### Statistical analysis

Statistical analysis and data plotting were carried out using the open access software R (version 4.1.2, 2021-11-01, “Bird Hippie”). The libraries employed were: tidyverse, readxl, dplyr, rstatix, ggpubr, ggplot2, plotrix, and magrittr. Two groups of experiments were carried out in this study. The first evaluated the production of quercetin nanocrystals at increasing HPMC concentrations, while the second evaluated three different types of polymeric stabilizer. Nanocrystal mean size and PDI were selected as responses. All statistical analyses and assumption tests were performed on both size and PDI. Data frames were checked for outliers, by constructing boxplots of the data distribution. Data points were grouped by sample (three replicates) and values were regarded as outliers if they were <(Q1-1.5 × IQR) or >(Q3 + 1.5 × IQR), where Q1 is the 25th percentile, Q3 the 75th percentile, and IQR the interquartile range.

Residual analysis was performed to test for the assumptions of the three-way ANOVA. Normality was assessed using the Shapiro–Wilk's normality test and homogeneity of variances was assessed by Levene's test. Residuals were considered normally distributed for *p*-values > 0.05, and homogeneity of variances was also assumed for *p*-values > 0.05. Three-way ANOVA was then carried out on the data, considering RCF (2 levels), RIAC configuration (2 levels), HPMC concentration (3 levels), or stabilizer type (3 levels) as independent variables. After three-way ANOVA was performed, post hoc tests were run to pinpoint differences between groups. Pairwise *t*-student tests were carried out between different groups of datasets. Differences were considered statistically significant for a Bonferroni adjusted *p*-value < 0.05. A full list of data, including residuals analysis and ANOVA results, is reported in the ESI† section.

## Results and discussion

### Preliminary tests: suitability of the production method and formulation development

In order to identify a suitable nanocrystal production method, different operational and formulation-related parameters were initially evaluated. Values for these parameters were either based on previous research demonstrating nanoparticle production using the RIAC (such as RCF and volume ratios between solvent and antisolvent)^46^ or on previous studies describing the production of quercetin nanocrystals (such as type and concentration of stabilizer).^25,32,56–59^. The centrifugation time needed to completely empty the reservoirs was evaluated first. 1 mL of DI water was placed in each reservoir and the reactor was run at different RCFs. At an RCF of 200, 9 minutes of continuous centrifugation were needed to empty the reservoirs; decreasing the RCF < 200 caused the emptying to become inconsistent and often incomplete. It was therefore decided that the RIAC should not be operated below this limit. RIACs emptying times at increasing RCF are reported in Table 1.

**Table 1:** RIACs emptying time (in minutes) for different values of RCF

RCF	500	750	1000	2000	3000	4000
Emptying time (min)	8	7	6	4	3	2

Following these initial tests, the production of quercetin nanocrystals was evaluated. The antisolvent medium was an aqueous solution of Kolliphor P 407 (KP407) at different concentrations (1%, 2% and 4% w/v). A range of RCF values were investigated, and the centrifugation time was set to the value needed to fully empty the reservoirs (see Table 1). In these preliminary tests, it was also assessed whether the RIAC configuration used had an effect on nanocrystal dimensions. The outcomes from these tests (in terms of particle mean diameter and PDI) and the corresponding experimental settings, are reported in Table 2.

**Table 2:** Summary of preliminary tests carried out to evaluate production of quercetin nanocrystals using the RIAC. Different particle stabilizers were employed, including KP407 (1%, 2% and 4% w/v), KP188 (1% and 4% w/v), and TW20 (1% and 4% w/v). Both spiral- and straight-RIACs were used and were operated at two different RCFs (500 to 2000). Nanocrystal mean diameter and PDI are reported for each of the experiments performed. When the produced suspension included coarse crystals, the DLS instrument gave a diameter of 10 000 nm and a PDI of 1

Stabilizer concentration	RIAC configuration	RCF	Peak mean diameter (nm)	PDI
KP407_1	SPIRAL	750	10 000	1
KP407_1	STRAIGHT	750	10 000	1
KP407_1	STRAIGHT	2000	10 000	1
KP407_2	STRAIGHT	500	10 000	1
KP407_4	SPIRAL	500	21.13	0.089
KP407_4	SPIRAL	1000	20.78	0.085
KP407_4	SPIRAL	2000	20.93	0.065
KP407_4	STRAIGHT	500	23.47	0.097
KP407_4	STRAIGHT	2000	20.91	0.074
KP407_4	STRAIGHT	2000	21.53	0.184
KP188_1	SPIRAL	750	10 000	1
KP188_1	STRAIGHT	750	10 000	1
KP188_4	SPIRAL	200	10 000	1
KP188_4	SPIRAL	2000	10 000	1
KP188_4	STRAIGHT	200	10 000	1
KP188_4	STRAIGHT	2000	10 000	1
TW20_1	STRAIGHT	2000	10 000	1
TW20_1	STRAIGHT	2000	10 000	1
TW20_4	STRAIGHT	2000	8.391	0.253

**Fig. 3:**
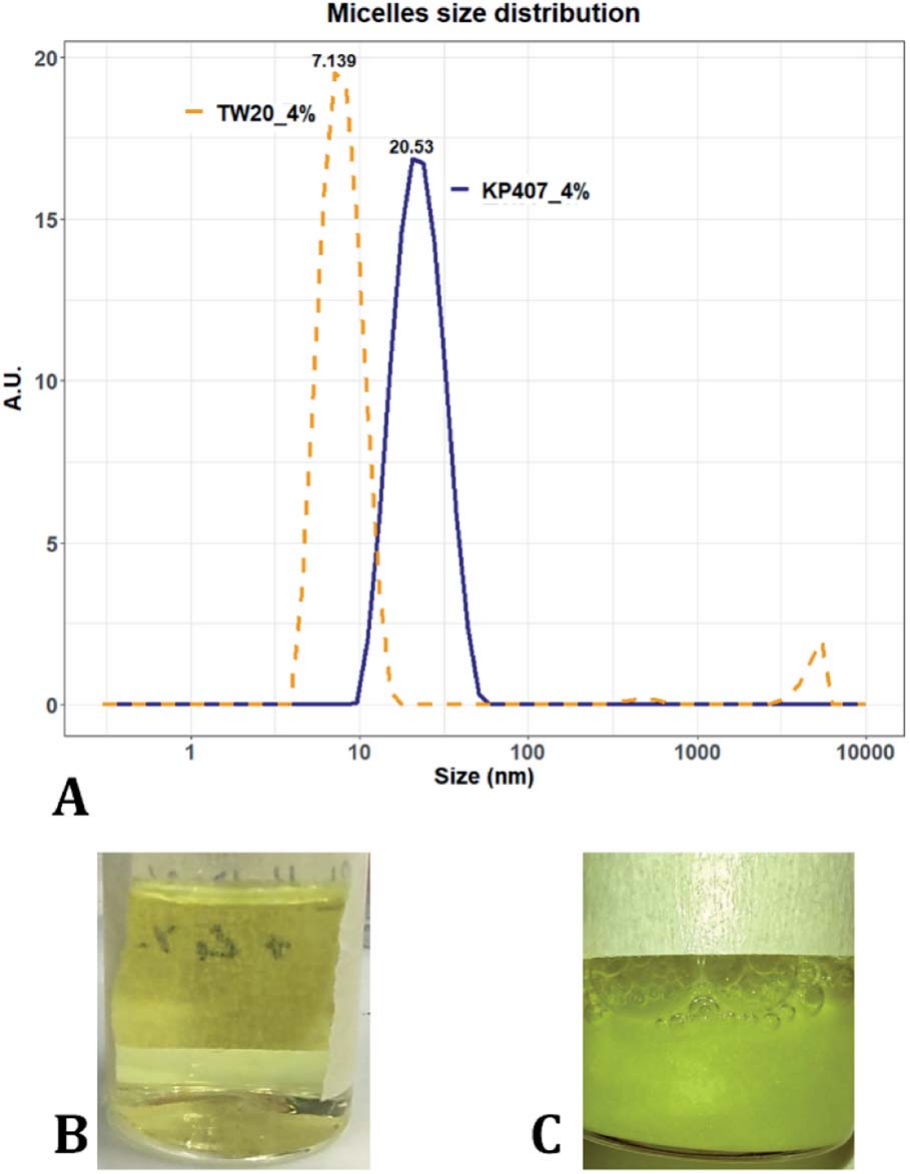
(A) Size distribution of samples manufactured using 4% KP407 (in blue) and 4% TW20 (in orange). These samples were optically clear and likely contained quercetin solubilised within polymeric micelles. (B) Photograph of a sample prepared using 4% KP407. Quercetin did not precipitate in the form of crystals or nanoparticles, as it was likely solubilised within polymeric micelles. (C) Photograph of a sample prepared using 1% KP407. Quercetin precipitated in the form of coarse acicular crystals that sedimented at the bottom of the vial.

Many of the samples produced in these tests consisted of a suspension of coarse quercetin acicular crystals, with large size dispersity. In all these cases the DLS instrument did not provide a reliable estimate of crystal size, but instead returned a value of peak mean diameter of 10 000 nm and PDI of 1 (Table 2). A representative image demonstrating these characteristics, is reported in Fig. 3C. For all RCF values and RIAC configurations tested with KP407 at a concentration of 1% or 2%, coarse crystals formed and precipitated rapidly. Increasing KP407 concentration to 4% resulted in samples that were optically clear (as shown in Fig. 3B), and contained nanoparticles with mean diameter < 22 nm and PDI between 0.065 and 0.184 (Table 2). A representative size distribution for these samples is reported in Fig. 3A (blue line). The dimension of nanoparticles in the presence of KP407 4% was not affected by either RCF or the RIAC configuration used. The stabilisers Kolliphor P 188 (KP188) and Polysorbate 20 (TW20) were subsequently tested, at concentrations of 1% and 4%. At both concentrations KP188 produced crystals that were large and had large size dispersity, using both RIAC configurations and at all RCF values investigated (Table 2). Crystals sedimented and aggregated rapidly upon production. Neither the RIAC architecture nor centrifugal force affected the properties of the obtained crystals. TW20 was evaluated using only the straight-RIAC at 2000 RCF (at both 1% and 4% w/v concentrations). As seen with KP407, when TW20 was used at 4% the obtained sample appeared optically clear and contained nanoparticles, with a mean diameter < 9 nm and PDI of 0.253. A representative size distribution for this sample is shown in Fig. 3A (orange line).

Despite KP407 4% and TW20 4% producing particles with a small size and low size dispersity, it was deemed unlikely that these particles corresponded to nanocrystals. This is largely because API nanocrystals with diameter < 50 nm, are substantially unreported in the literature.^64–67^ Consistent with previous investigations,^68–70^ it is likely that the produced samples contained polymeric micelles capable of solubilising quercetin in the formulation, thus preventing it from precipitating and forming nanocrystals. This hypothesis is supported by data from the literature,^69,70^ reporting on the solubilization of quercetin in both KP407 and Pluronic P123 micelles. The diameter of quercetin-loaded KP407 micelles in these previous studies was 28.79 ± 0.8 nm, which is comparable to the results reported here. To confirm that KP407 alone could form micelles using the RIAC, a ‘blank’ sample was produced, using the same amount of KP407 without quercetin. As reported in the ESI (Fig. S1 and S2),† KP407 formed micelles that were 6.44 ± 0.18 nm in diameter. The difference in diameter compared to micelles obtained in the presence of quercetin, could be attributed to the incorporation of quercetin molecules within the micellar structure. On the other hand, TW20 formed micelles that were 7.60 ± 0.08 nm in diameter and were comparable in size to those obtained in the presence of quercetin.

The RIAC has proven effective at inducing rapid mixing between chemical species,^46^ which is beneficial for achieving uniform crystal nucleation. The preliminary findings of this work however, suggest that the crystal growth process requires further optimization. According to the nucleation and growth theory, a diffusion-limited growth would favour controlled formation of quercetin nanocrystals by nanoprecipitation, which results in a particle suspension of smaller mean size and reduced size dispersity.^34^ It was hypothesised that increasing the viscosity of the medium would impact positively on the nanoparticle formation process, allowing for a final product of superior quality. Due to the increased viscosity of the medium, and the consequent reduction in quercetin diffusion rate, the crystals growth process would be limited by the diffusion of quercetin rather than by the rate of deposition of new drug molecules on the surface of the forming particles (referred to as ‘surface mechanism’ in the literature).^25,32,71^ One of the most commonly used viscosity enhancers in API nanocrystal production is HPMC.^72–75^ The addition of HPMC to the formulation containing KP407 1%, at a concentration of 0.5% w/v was thus explored. Preliminary results showed the produced sample was slightly opaque, with mean particle diameter < 200 nm. A second formulation with KP407 at 4% was subsequently tested, but this again yielded micellar solubilization of quercetin. This confirmed that greater stabilizer concentration prevents nuclei formation and hinders the precipitation process.

### Considerations on the manufacturing of quercetin nanocrystals using the RIAC

The mechanism of nanocrystal formation using the RIAC, is believed to be concurrent to those already described for classical microfluidic devices.^25,36,37^ Nanocrystals are specifically manufactured through controlled precipitation, and the formation mechanism is described by the LaMer theory.^32,33^ Consistently with chip-based microfluidic methods, in the RIAC a solution of the API (in an organic solvent) mixes with an aqueous solution (antisolvent), which in turn initiates the particle formation process. The RIAC relies on centrifugal forces to drive liquids from its reservoirs into the mixing channel, where rapid and controlled mixing of solvent and antisolvent occurs. As reported previously for comparable flow-through reactors, the fluid viscosity, volumetric flow rate, flow rate ratio between solvent and antisolvent, channel diameter and geometry, will all influence flow and mixing regimes within the reactor.^76,77^ Mixing of chemical species can occur by either diffusion or advection, whereby diffusion is driven by the concentration gradient of chemical species whilst advection is the transport of species by bulk motion of a liquid. Due to the geometry of the mixing channel in the spiral-RIAC, formation of secondary vortical flows is likely to occur,^76^ resulting in advection-dominated mixing. The latter form of mixing is known to be more rapid and effective when compared to diffusion. Increasing the volumetric flow rate and mixing channel's diameter, length, and radius of curvature is expected to increase mixing efficiency, whilst increasing fluid viscosity is anticipated to have the opposite effect.

On the other hand, the straight-RIAC would theoretically allow for the formation of a central stream of quercetin solution, laterally focused by two laminar streams of polymer aqueous solution. Transport of solvent and antisolvent in this case is expected to occur predominately by diffusion. Increasing the volumetric flow rate, fluid viscosity, and mixing channel's diameter is expected to reduce the mixing efficiency, whilst increasing the channel length would have the opposite effect. It should be noted that, since it was not possible to visually inspect the mixing channel during operation of the RIACs, the corresponding mixing regimes could not be confirmed experimentally.

**Fig. 4:**
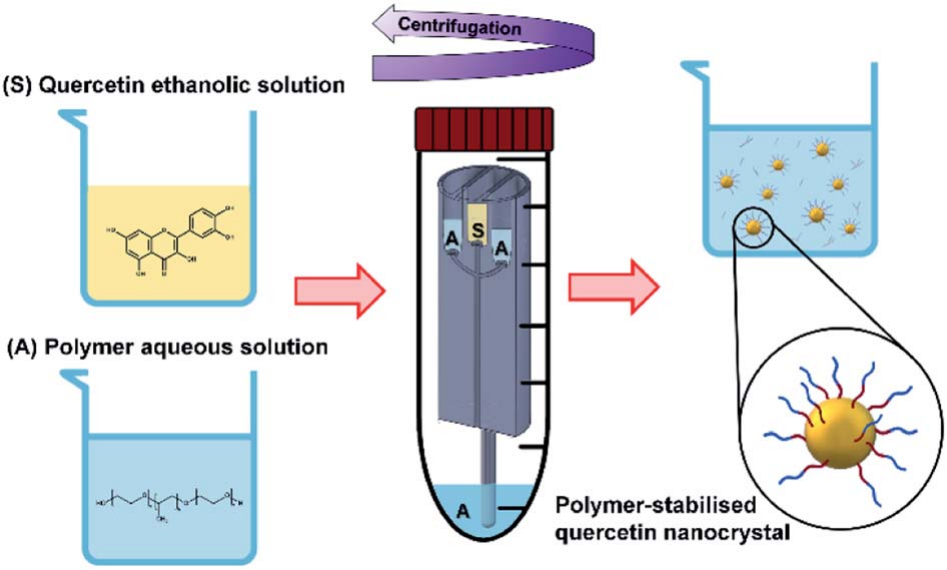
In the RIAC, nanoparticles are produced in a similar way to pump-driven microfluidic devices that rely on the mixing between a solvent and an antisolvent. The quercetin organic solution (S) and the aqueous solution containing an amphoteric polymer (A) are first placed in the RIAC reservoirs and at the bottom of the centrifuge tube. When the reactor is actuated inside the centrifuge, S and A flow from the reservoirs into the mixing channel, where rapid and controlled mixing occurs. In this process, the aqueous solution acts as the antisolvent and allows the precipitation of the drug. The newly formed nanoparticles exhibit high surface tension and the amphoteric polymer acts as a stabilizer, covering the nanocrystals surface and hindering interparticle aggregation.

When the mixing of solvent and antisolvent occurs, quercetin enters a supersaturation state that initiates the nucleation process and results in the formation of fine quercetin nanoparticles.^32,33^ Since quercetin is a highly hydrophobic molecule, the newly formed nanoparticles exhibit high surface tension. To prevent interparticle aggregation, amphoteric molecules are added to the aqueous solution. According to the DLVO theory,^78–80^ many factors play a role in determining the degree of nanoparticle surface coverage by the stabilizing molecule. These include the chemical properties of stabilizer and nanoparticles, mixing efficiency of the reactor, absorption kinetics of the polymer onto the nanoparticles, and the relative concentration of polymer and nanoparticles. Overall, the protocol conceived in this study aimed to achieve rapid and efficient mixing of the reagents, favouring homogeneous quercetin precipitation and effective nanoparticle surface coverage by the stabilizers.^22,81^ A schematic representation of quercetin nanocrystals formation and their surface coverage by the amphoteric stabilizer is illustrated in Fig. 4.

Since the preliminary tests described above showed that increasing the medium viscosity favoured nanocrystals formation, HPMC was employed as a viscosity enhancer in all subsequent experiments, with the concentration of polymer stabilizer (KP407, KP188 or TW20) set at 1% w/v. RCF values were also kept above the limit of 200, and 5 mL of suspension (corresponding to a final quercetin concentration of 0.9 mg mL^−1^) were produced using either type of RIAC. The experimental plan was then divided into two groups. The first aimed at evaluating the effect of varying the medium viscosity while the second evaluated different types of stabilizers. In both cases, the effect of changing RCF (500 or 3000) and the RIAC configuration (spiral or straight) were also assessed.

### Effect of varying the viscosity of the medium

**Fig. 5:**
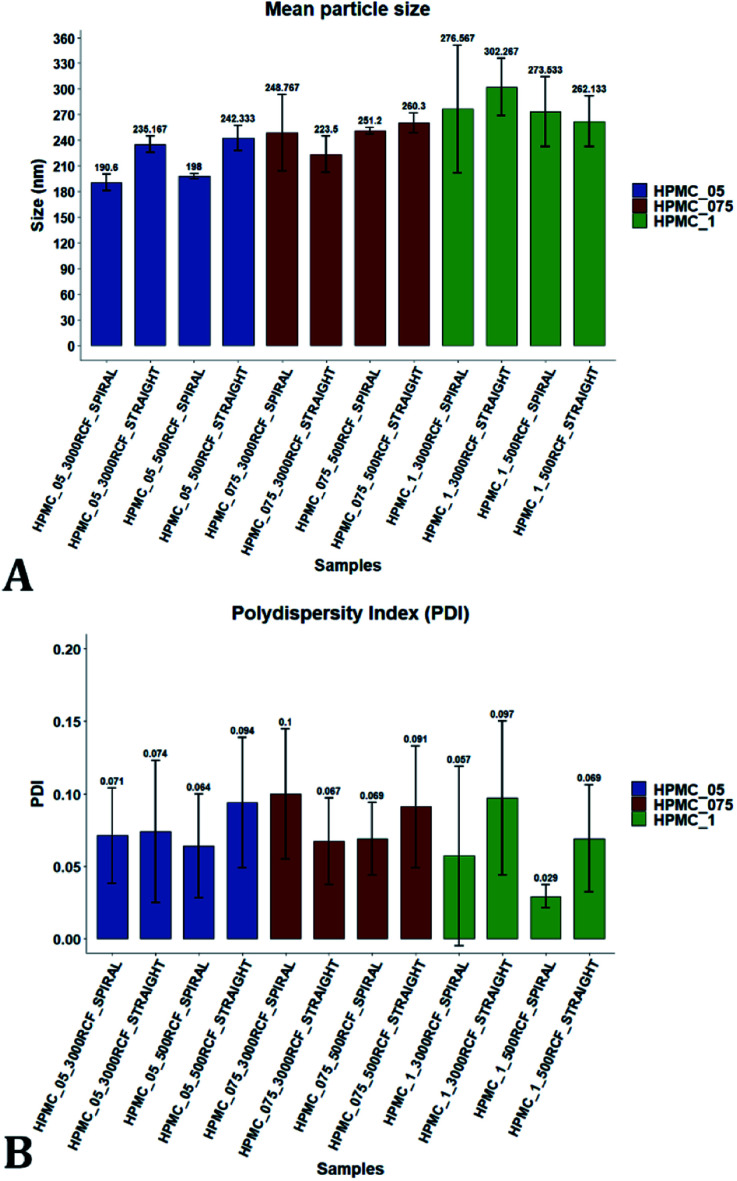
Mean particle size (A) and PDI (B) of quercetin nanocrystals manufactured using the two different RIAC architectures. Samples were prepared in triplicate, using both RIAC configurations operated at two RCF levels (500 and 3000). Samples were prepared using three different concentrations of HPMC, corresponding to 0.5%, 0.75% and 1% (in blue, red and green respectively). Nanocrystal size and RSD of triplicates increased with increasing HPMC concentration, whereas PDI remained relatively low and almost unchanged throughout all samples.

Variation of the viscosity of the medium was evaluated, to determine its impact on nanocrystal properties. The underlying hypothesis was that including a viscosity enhancer in the formulation, would reduce the diffusion rate of quercetin in the medium, resulting in diffusion-limited crystal growth. According to the LaMer theory,^32,33^ this is desirable to obtain particles with small size and low size dispersity. Increased medium viscosity could also impact on mixing efficiency within the RIAC, which would affect the size distribution of the resulting particles. Varying amounts of HPMC were investigated, to determine the effect on nanoparticle mean diameter. 1% (w/v) KP407 was used as stabilizer solution for each sample, which was produced and analysed in triplicate. The mean size and PDI of the obtained quercetin nanoparticles are shown in Fig. 5, where samples are coloured by HPMC concentration (0.5%, 0.75%, and 1% w/v). For each HPMC concentration, the RIAC was operated at RCF values of 500 and 3000 using both RIAC configurations. A total of four experimental conditions per HPMC concentration tested were therefore employed.

As shown in Fig. 5, high manufacturing repeatability was demonstrated using the RIACs, which is also reflected in the small standard deviation of mean particle size and the absence of outliers in the data frame. Notably, increasing medium viscosity led to an increase in mean particle size. The mean particle diameter for samples manufactured using 0.5%, 0.75% and 1% HPMC, was respectively 216.53 ± 26.00 nm, 245.942 ± 15.76 nm and 278.63 ± 16.94 nm. However, a greater standard deviation was observed between replicates. The mean relative standard deviation (RSD) for the four samples manufactured using 0.5%, 0.75% and 1% HPMC was respectively 4.167 ± 1.987%, 8.406 ± 7.225%, and 16.011 ± 7.492%.

All samples produced had very low size dispersity, as none of the measured PDI values was greater than 0.14 (Fig. 5). In addition, there appeared to be no clear dependence of PDI on the medium viscosity used. To evaluate any significant difference between samples' mean particle size and PDI, a three-way ANOVA was performed followed by post-hoc *t*-test pairwise comparisons on the data. The complete dataset is reported in the ESI (Tables S1–S4).† When a three-way ANOVA was performed using PDI as response, no main effect or interaction was found to be significant. When particle mean diameter was used as response, no three-way or two-way interaction was found to be significant, but the main effect ‘HPMC concentration’ was statistically significant (*p* = <0.001). This confirmed that varying medium viscosity had an impact on the mean size of quercetin nanocrystals. Increased particle size with increasing the medium viscosity was expected, as greater viscosity corresponds to lower quercetin diffusivity and slower mixing within the reactor. Both of these effects may in turn result in slower nuclei formation and particle growth, leading to the formation of nanocrystals with larger mean diameter and a broader size distribution.^32^

**Fig. 6:**
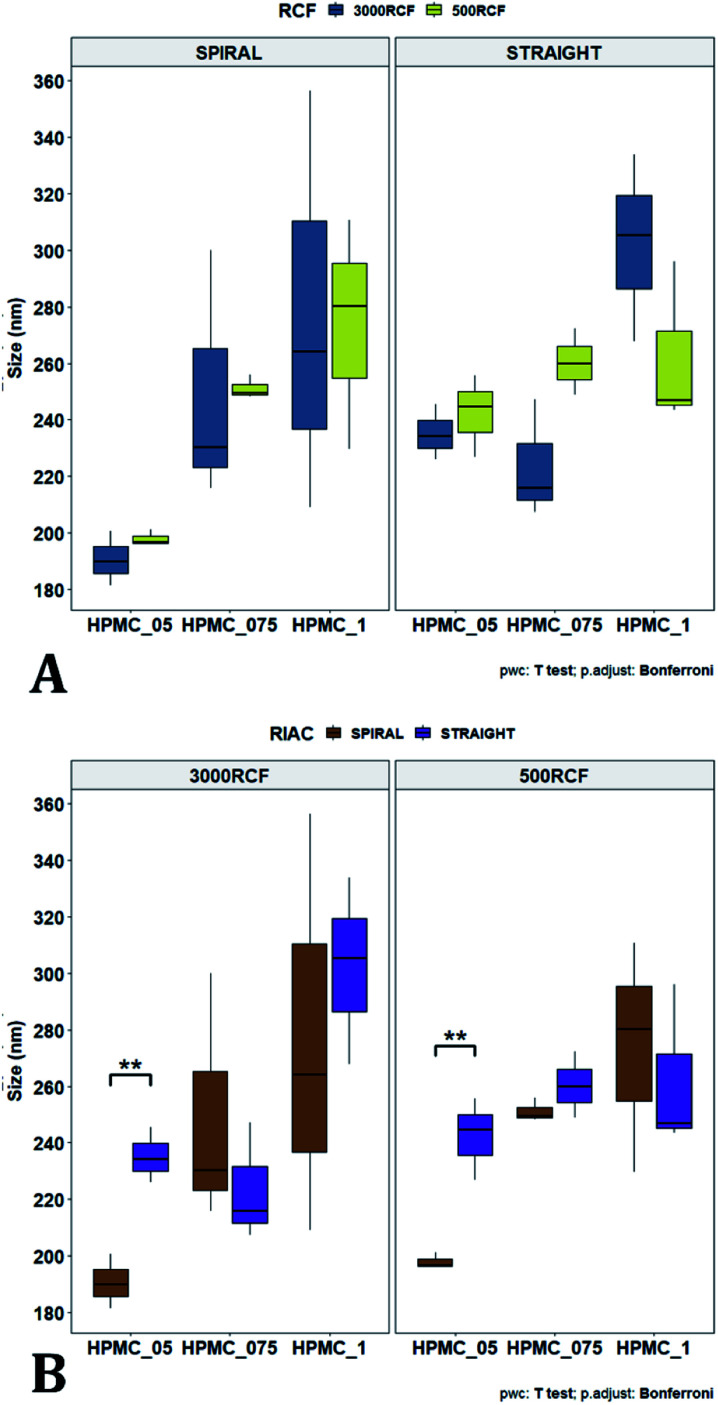
Mean particle size comparisons between samples manufactured at the same HPMC concentration. Plots highlight differences in particle size determined by changes in (A) RCF and (B) RIAC configuration used. (A) Comparison of particle size for samples prepared at 500 RCF (in yellow) and 3000 RCF (in blue). Nanoparticles didn't show significant differences at any HPMC concentration level (0.5%, 0.75% and 1% w/v). (B) When samples were prepared using different RIAC architectures (straight-RIAC in purple, and spiral-RIAC in brown) significant differences were detected between samples prepared using 0.5% HPMC, at both 500 and 3000 RCF (*p* < 0.005 in both cases).

**Fig. 7:**
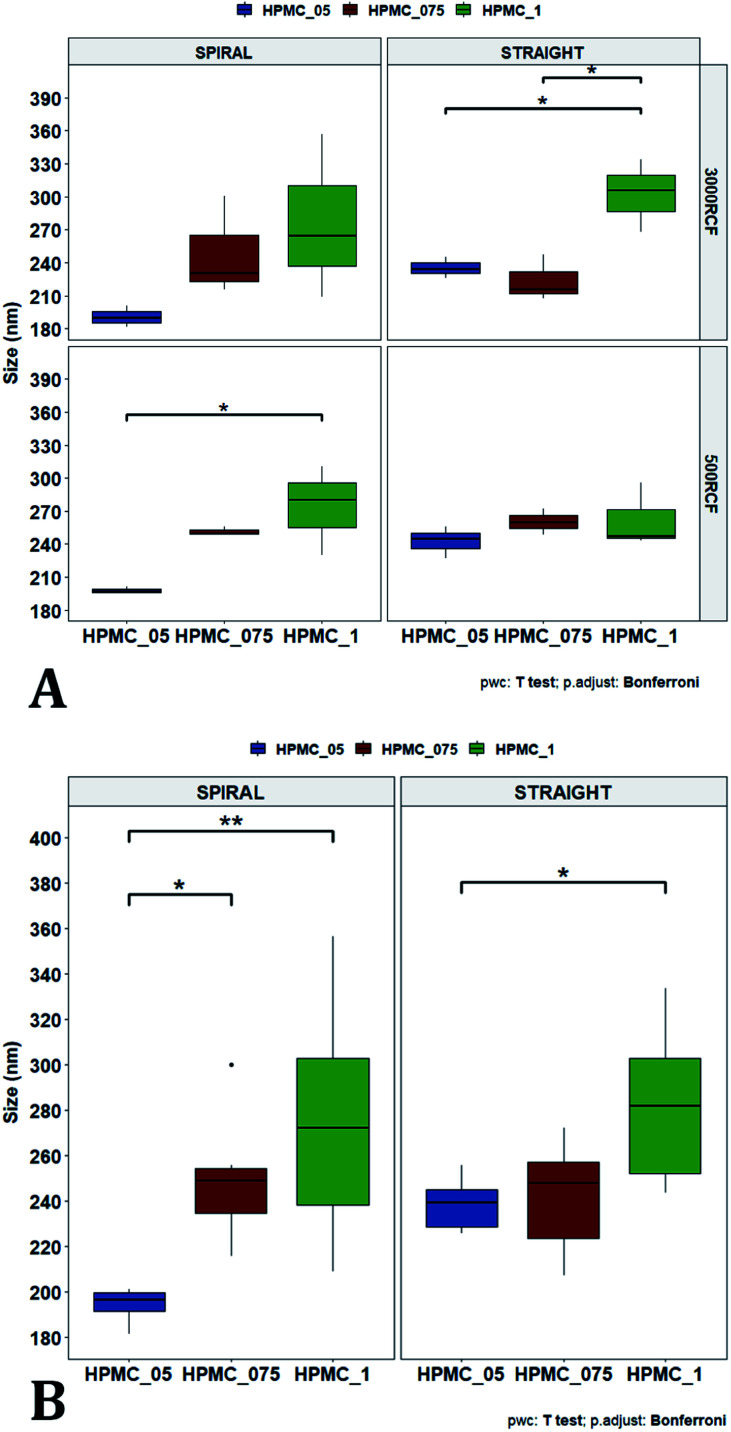
Mean particle size comparisons between samples manufactured using increasing HPMC concentration (0.5%, 0.75% and 1%). Plots highlight differences in nanocrystal mean diameter determined by changes in HPMC concentration. Panel (A) shows differences in particle mean diameter due to both the variation of RIAC architecture and RCF value used. For samples prepared using the spiral-RIAC, there is a clear increase in mean particle diameter when HPMC concentration is increased (at both RCF levels tested), although only samples prepared using 0.5% and 1% HPMC at 500 RCF are significantly different (*p* < 0.05). Concerning the straight-RIAC, samples prepared using 0.5% and 0.75% HPMC appear very similar to each other at both RCF levels. The sample prepared using 1% HPMC shows a significant increase in particle diameter only when the production is conducted at 3000 RCF. The plot in panel (B) groups the samples prepared at different RCFs and illustrates differences in mean particle size due to the variation of HPMC concentration (for both RIAC architectures employed). An increase in particle size with increasing HPMC concentration can be appreciated.

To pinpoint specific significant differences in the data frame, different pairwise *t*-tests were performed on specific groups of data (results are shown in Fig. 6 and 7). In Fig. 6, the effects of changing RCF or RIAC configuration were analysed. Concerning the effect of RCF, no significant difference between samples prepared with the same RIAC and the same HPMC concentration was detected (Fig. 6A). Changing RCF would impact on the total flow rate (TFR) through the mixing channel, which in turn influences both residence time of chemical species within the device and mixing velocity, whilst the volume ratio between solvent and antisolvent remains unchanged. A number of previous studies concluded that the TFR within flow reactors has a significant effect on nanoparticle size.^25,82–85^ In some cases, increasing TFR led to a reduction in nanoparticle size,^86^ whilst in other studies no significant effect of TFR was determined.^87,88^ Since results from this study do not show significant differences in production performance between 500 and 3000 RCF, it could be inferred that although changes in TFR and residence time are apparent, complete mixing is likely achieved at both of these RCF values.

This behaviour is observed for both RIAC configurations, suggesting that complete mixing likely occurred in both device prototypes, even though they may present different mixing regimes (as discussed above). The effect of using different RIAC configurations on nanocrystal dimensions was then evaluated in greater depth, for all different HPMC concentrations used (see Fig. 6B). The results confirm that the performance of both RIACs is largely comparable across most conditions investigated (RCF and HPMC concentrations). A statistically significant difference in nanocrystal size between the two RIACs was detected only for HPMC of 0.5%, at both 500 and 3000 RCF. In these cases, the spiral-RIAC led to a smaller nanoparticle diameter (190.60 ± 9.73 nm and 198.00 ± 2.79 nm) compared to the straight-RIAC (235.17 ± 9.77 nm and 242.33 ± 14.53 nm). As discussed earlier, the mixing channel architecture of the spiral-RIAC is likely to induce faster mixing compared to the straight-RIAC. Secondary vortical flows (also known as Dean flows) are known to form within curved channels, inducing advection-dominated transport and enhancing mixing efficiency.^76,77^ It could therefore be inferred that mixing in the spiral-RIAC is likely to be advection-dominated, whilst in the straight-RIAC it may occur predominately by diffusion. Faster mixing in the spiral-RIAC could also be attributed to the longer mixing channel, hence the greater residence time, compared to the straight-RIAC. The rapidity of mixing is expected to further increase at the lower HPMC concentration used (0.5%), given the lower medium viscosity. This would explain the observed difference between RIAC configurations at 0.5% HPMC. Faster mixing has been previously associated with the formation of smaller nanoparticles, in studies using microfluidic-based devices that relied on comparable mechanisms of particle formation.^25,82,87,89^ A non-linear relationship may exist between mixing efficiency and HPMC concentration, which renders differences in performance between RIACs negligible at HPMC concentrations > 0.5%. For instance, increasing the medium viscosity (at a given RCF) reduces the flow Reynolds number in the mixing channel, which may potentially suppress secondary flows (or reduce their strength)^76,90^ and render the spiral-RIAC closer to a diffusion-dominated mixer. Further characterization of mixing performance in both RIACs (*i.e.*, using numerical simulations) should be carried out in the future to gain a more pervasive understanding of the mixing process in these systems.

In Fig. 7A, samples were grouped by RCF and RIAC configuration, and plotted against the three HPMC concentrations used. Concerning the spiral-RIAC, there is a clear trend for nanoparticle size to increase as HPMC concentration increases (from 190.60 ± 9.73 nm to 276.57 ± 74.43 nm), even though only one pair of samples was significantly different. This trend is apparent at both RCF values. The straight-RIAC instead showed different outcomes. At 500 RCF, HPMC concentration seems to have little to no effect on nanocrystal size, while at 3000 RCF there is a statistically significant difference in particle diameter between samples prepared using 1% HPMC and those prepared with 0.5% and 0.75% HPMC. However, these two latter samples did not show a statistically significant difference. Discrepancies in behaviour between the two types of RIAC could be attributed to the specific mixing regimes within these systems, as discussed earlier. The spiral-RIAC shows a non-linear dependence of nanoparticle size on HPMC concentration, which may be due to reduced mixing rapidity with increasing medium viscosity. The straight-RIAC instead appears to have comparable mixing performance across all HPMC concentrations at 500 RCF, as well as between 0.5% and 0.75% HPMC at 3000 RCF. The larger particle diameter obtained at the greatest viscosity evaluated (corresponding to 1% HPMC) and 3000 RCF may suggest that mixing at these conditions is less rapid compared to the other experimental conditions evaluated. Assuming that the straight-RIAC operates on the basis of diffusion-dominated mixing (as for conventional microfluidic hydrodynamic flow focusing devices), increasing both TFR and medium viscosity contributes towards slowing down mixing between solvent and antisolvent, which in turn may allow for the formation of larger particles. This has been previously reported for other types of nanoparticulate drug delivery systems produced using flow reactors.^25,82,87^

Finally, Fig. 7B illustrates a comparison between samples grouped by RIAC configuration. In this representation, the comparison between samples obtained at different RCFs cannot be appreciated, but results described above (see Fig. 6A) demonstrate a marginal effect of RCF on nanocrystal size. This plot further confirms that HPMC concentration impacts on nanocrystal size. The effect is more apparent when the spiral-RIAC is used, as nanoparticle size increases with medium viscosity, although samples at 0.75% and 1% HPMC are not statistically different (which may be due to increased end-product variability at the greater viscosities used). Conversely, the straight-RIAC is less sensitive to medium viscosity, although it displays a statistically significant difference in nanoparticle size between samples prepared with 0.5% and 1% HPMC.

### Effect of varying the type of stabilizer

**Fig. 8:**
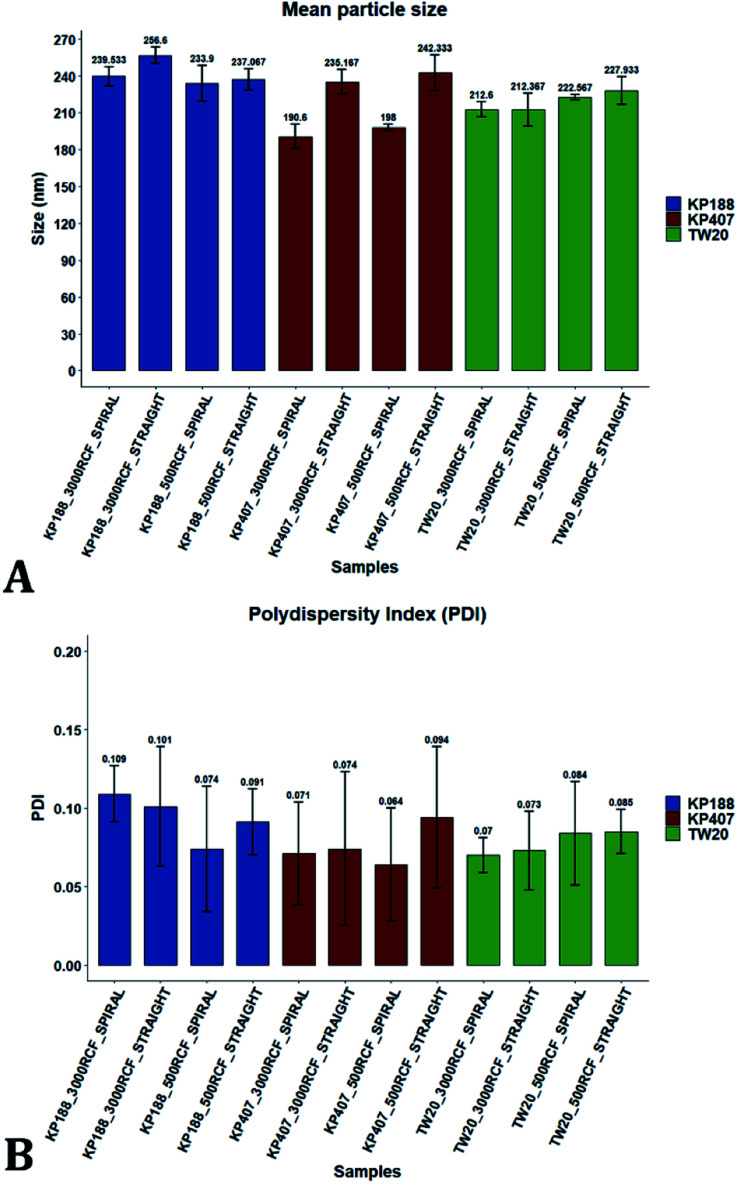
Mean particle size (A) and PDI (B) of quercetin nanocrystals manufactured using the two different RIAC architectures. Samples were prepared in triplicate, and both RIAC configurations were operated at two RCF levels (500 and 3000). Samples were prepared using three different polymeric stabilizers: Kolliphor P 188 (KP188), Kolliphor P 407 (KP407) and polysorbate 20 (TW20) (in blue, red and green, respectively). Nanocrystals size underwent appreciable variations when the stabilizer was changed, whereas PDI remained quite low and almost unchanged throughout all samples.

After evaluating the effect of medium viscosity, a subsequent series of experiments investigated the potential effect of changing the type of polymeric stabilizer on nanocrystal dimensions. For these tests three stabilizers were selected, Kolliphor P 188 (KP188), Kolliphor P 407 (KP407), and Tween 20 (TW20). All of the stabilizers have low toxicity and are widely accepted pharmaceutical excipients.^91,92^ There are two main mechanisms through which a colloidal suspension can be stabilized: electrostatic repulsion and steric stabilization,^93,94^ which rely on the use of ionic and non-ionic stabilizers, respectively. Polymeric stabilizers promoting steric stabilization as used in this work, are composed of two functional moieties: (i) an anchoring tail segment that interacts with the nanoparticle and allows polymer absorption onto the particle surface, and (ii) another tail segment that undergoes solvation. Solvation of this segment is necessary to achieve a stabilizing effect, as it thermodynamically prevents interpenetration of polymer chains when two colloidal particles approach each other. For this reason, if the stabilizer tail can be solvated efficiently by the chosen medium, steric stabilization is usually capable of preventing particle aggregation. A limitation associated with steric stabilisation of nanoparticles is the need to finely tailor the anchoring tail to the drug of interest. Due to the lack of fundamental understanding of the interaction mechanisms between the stabilizer and nanoparticle surface, current stabilizer screening approaches are mostly empirical.^92,95,96^ Two different grades of poloxamer were assessed; the more lipophilic (KP407) and the more hydrophilic (KP188). Both KP188 and KP407 had been used in previous work for quercetin nanocrystal production with successful outcomes.^97–99^ Polysorbate 20 (TW20) was utilised as a stabilizer to verify whether differences in the chemical structure between stabilizers, could have an impact on nanocrystal production. Previous research has shown that varying the stabilizer formulation can lead to changes in particle size, size dispersity, and physical stability.^92,95,97–100^ Therefore, similar experimental outcomes were expected in the present study. The chosen stabilizers (KP188, KP407 and TW20) were all employed at a 1% w/v concentration in aqueous solution, which also contained 0.5% w/v of HPMC. Only 0.5% HPMC was used in the formulation as previous results (see Fig. 6) showed that increasing the HPMC concentration further led to greater sample-to-sample variability. RCF values of 500 and 3000 were used and tested in both RIAC configurations in triplicate. Fig. 8 shows the mean diameter and PDI of quercetin nanocrystals obtained in these experiments, where bars are coloured by stabilizer type. The production process was again highly repeatable, with RSD < 6.5% for all samples and no outlier detected. The PDI was also low for all samples (<0.14). PDI values appear largely unaffected by the parameter changes performed, whereas the nanoparticle size appears to be influenced by the type of stabilizer used during production.

**Fig. 9:**
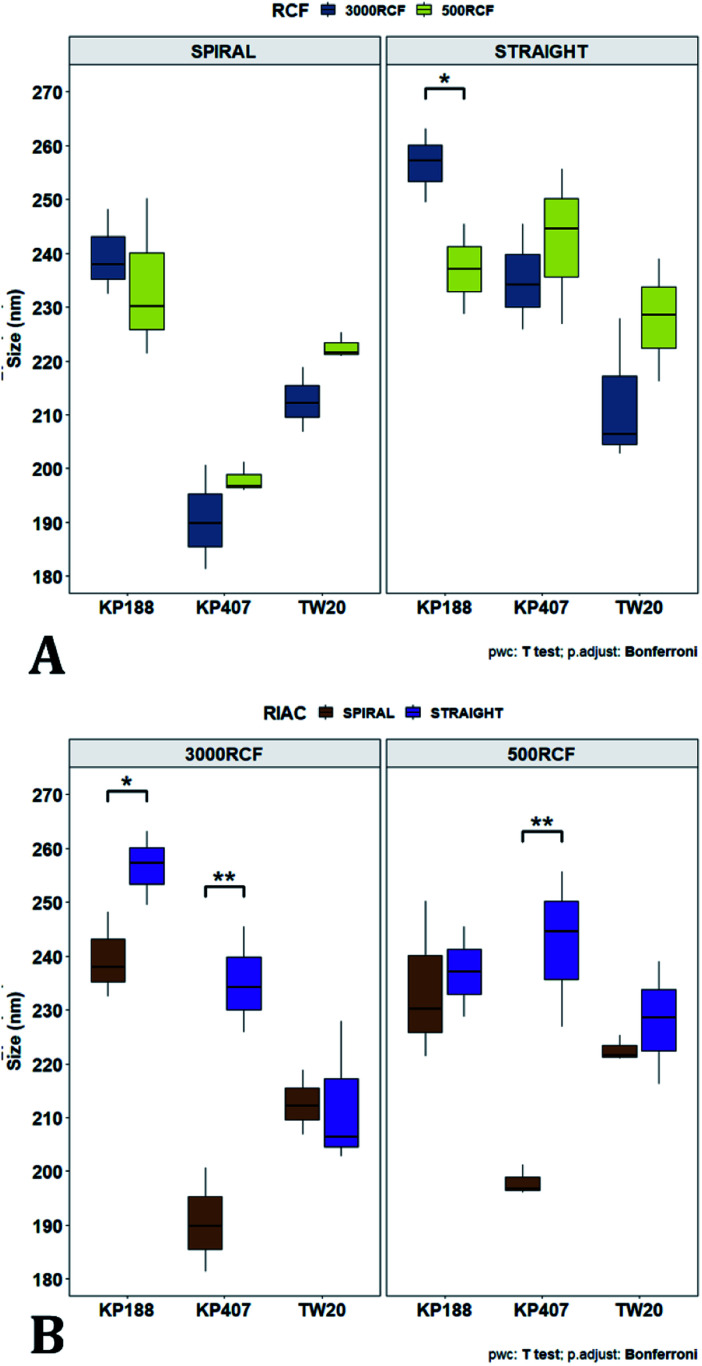
Mean particle size comparisons between samples manufactured using the same polymeric stabilizer. Plots highlight differences in nanoparticle size determined by changes in (A) RCF and (B) RIAC architecture. (A) Only samples prepared using Kolliphor P 188 (KP188) and the straight-RIAC showed significant particle size differences due to changes in RCF (*p* < 0.05). (B) When samples were prepared using different RIAC architectures (straight-RIAC in purple and spiral-RIAC in brown) significant differences in size were found between samples prepared using Kolliphor P 407 (KP407) as stabilizer, at both 500 and 3000 RCF (*p* < 0.005), and using KP188 (but only at 3000 RCF; *p* < 0.05).

**Fig. 10:**
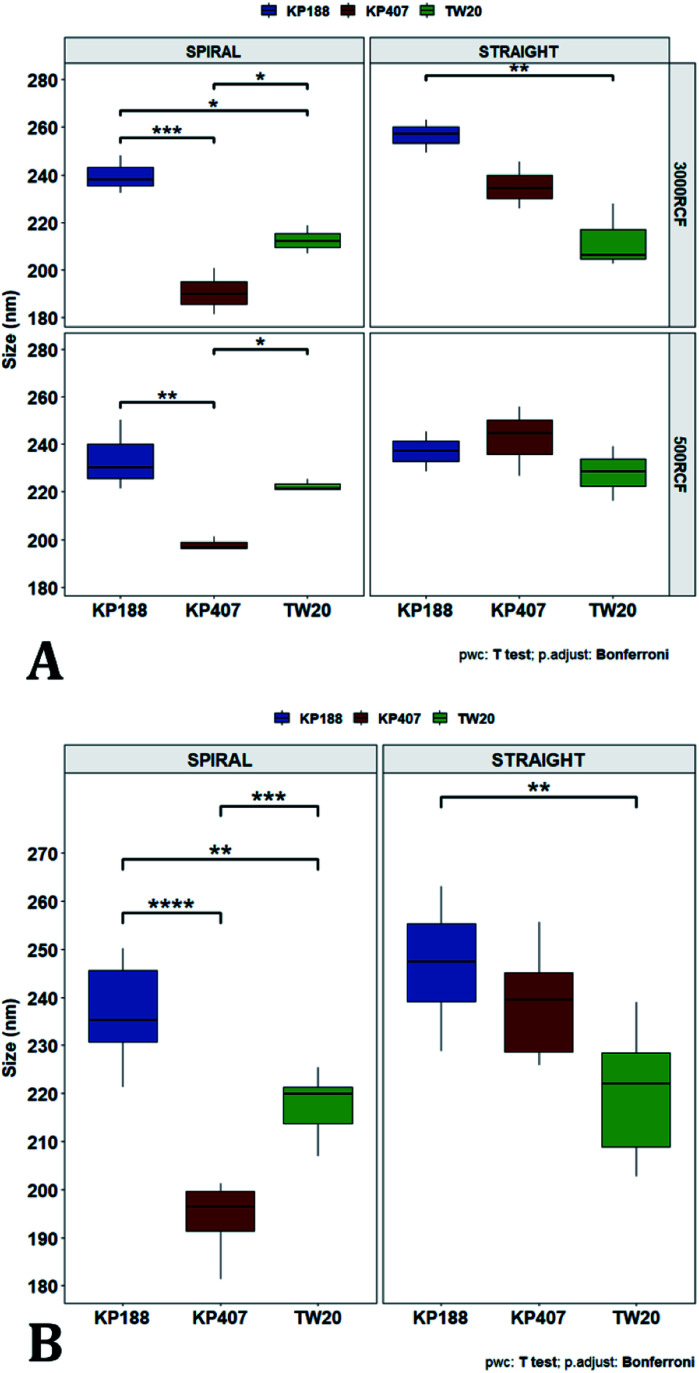
Mean particle size comparisons between samples manufactured using different polymeric stabilizers: Kolliphor P 188 (KP188), Kolliphor P 407 (KP407) and polysorbate 20 (TW20). Plots highlight differences in nanocrystal mean diameter determined by changes in the stabilizer used. The top panel (A) shows differences in particle mean diameter due to both the variation of RIAC architecture and RCF used for the production of the samples. For samples prepared using the spiral-RIAC, significant differences between most of the samples are found, as represented by the brackets (* = *p* < 0.05; ** = *p* < 0.005; *** = *p* < 0.0005). Concerning the straight-RIAC, samples prepared at 3000 RCF show appreciable differences, with a significant difference in size between samples prepared using KP188 and TW20. At 500 RCF, the manufactured samples do not show any significant difference. Panel (B) groups samples prepared at different RCFs and shows differences in mean particles size due to the change of stabilizer used (when the manufacturing is carried out using the two RIAC architectures).

The results of three-way ANOVA followed by *t*-test pairwise comparisons on the data were then analysed; the complete dataset is reported in the ESI section (Table S5, S6, S7 and S8).† When three-way ANOVA was performed using PDI as response, no main effect or interaction was found to be significant. This corroborates the findings reported earlier (shown in Fig. 5), confirming that PDI was largely unaffected by the tested parameters within the parametric space of this work. When three-way ANOVA was performed using particle mean diameter as response, no three-way interaction was found to be significant, while two two-way interactions and two main effects were significant (*p* < 0.05). Specifically, ‘RIAC configuration’ and ‘stabilizer type’ main effects were statistically significant (*p* = 5.49 × 10^−6^ and *p* = 1.80 × 10^−6^, respectively). The two-way interactions ‘stabilizer type-RIAC configuration’ and ‘stabilizer type-RCF’ were also statistically significant (*p* = 4.89 × 10^−5^ and *p* = 1.10 × 10^−2^, respectively). To pinpoint specific significant differences in the data frame, different pairwise *t*-tests were performed on the data (results are shown in Fig. 9 and 10). Fig. 9 illustrates the effect of changing RCF or RIAC architecture on quercetin nanocrystal mean diameter. Concerning the effect of RCF (Fig. 9A), only the sample prepared using KP188 showed significant changes in particle size when produced at different RCFs, but this was apparent only when the straight-RIAC was used. Despite this, results again support the observation that both RIACs are largely insensitive to changes in the actuating centrifugal force. Notably, this is consistent with previous research that used the RIAC to produce liposomes,^46^ where vesicle size was not influenced by RCF. Concerning the effect of RIAC configuration (Fig. 9B), a statistical difference in nanocrystal diameter between spiral- and straight-RIAC was observed in the presence of KP407 (at both 500 and 3000 RCF) and KP188 (at 3000 RCF).

In Fig. 10, samples were grouped by RCF and RIAC configuration and were plotted against the stabilizer type. When the spiral-RIAC is employed, differences between samples are evident. At 3000 RCF all samples are significantly different to each other; at 500 RCF, statistically significant differences are present between KP188 *vs.* KP407 and KP407 *vs.* TW20. Conversely, when nanoparticles are produced using the straight-RIAC, no significant effect of the stabilizer type is appreciated at 500 RCF, whereas at 3000 RCF, KP188 and TW20 are statistically different. Differences between the two RIAC configurations could be attributed to the different residence time of the chemical species inside the two reactors. The spiral-RIAC has a longer mixing channel and thus allows for greater residence time compared to the straight-RIAC. This could allow for more efficient absorption of polymers onto the nanoparticle surface. The effect of reducing the residence time would be more pronounced for polymers that have a slower kinetics of absorption. Although there is no available quantitative information about the absorption kinetics of the chosen polymers onto quercetin nanoparticles, it could be hypothesized that KP407 presents the slowest absorption kinetics, as differences between the two RIAC configurations are significant at both 500 and 3000 RCF. KP188 may instead have a faster absorption kinetics, as differences between the two RIACs are notable only when the residence time is increased to 3000 RCF. TW20 appears to have the fastest absorption kinetics, as no significant difference has been detected between experimental conditions tested. The plot in Fig. 10B shows the comparison between samples grouped by RIAC configuration. Whilst experiments have shown that nanoparticle size is almost insensitive to RCF (see Fig. 6B), the graph clearly shows that changing the stabilizer impacts on nanocrystal size. Consistent with the results shown in Fig. 9B, it appears as samples containing KP188 and TW20 undergo only minor dimensional changes, when production is performed with either spiral- or straight-RIAC. Conversely, samples prepared with KP407 have a different size distribution depending on the RIAC architecture used. It could be hypothesized that these differences are due to the different absorption kinetics of the polymers onto the nanoparticles surface. Other factors that may have impacted on particle size include differences between medium viscosity (which depend on the stabilizer used), affinity between quercetin and stabilizer monomers, and stabilizer diffusivity. It is difficult to ascertain which of these factors has the greater impact on the characteristics of the obtained nanoparticles. Overall, results show that the type of stabilizer plays an important role in determining the dimension of quercetin nanocrystals, whilst the effect of RIAC architecture is dependent upon the type of stabilizer used, with some formulations being less sensitive than others.

### Comparison with other production methods and nanoparticle stability issues

The results obtained in this work confirm that the RIAC is a promising method to achieve facile and rapid manufacturing of quercetin nanocrystals with low size dispersity and satisfying reproducibility.

In a previous work, Kakran *et al.* evaluated the production of quercetin nanocrystals with different methods (HPH, bead milling, and cavi-precipitation).^56^ All of the tested methods were successful, resulting in nanocrystals with size ranging from 276.7 nm to 787.3 nm and PDI between 0.111 and 0.238. The methods used however come with drawbacks when compared to the RIAC. Firstly, both HPH and cavi-precipitation necessitate a specialized high-pressure homogenizer, usually operated between 500 and 1500 bars. The processing time is variable, *i.e.*, around 60 minutes for cavi-precipitation and 20 processing cycles (time not defined) for HPH. The bead milling process suffers from similar disadvantages, as it relies on the use of a specialized bead milling instrument as well as milling beads. The processing time for this method is variable and depends on the hardness of the drug particles. All of these methods therefore necessitate highly specialized tools and relatively long processing times when compared to the RIAC, and the obtained nanoparticles are usually larger and with greater size dispersity. Unfortunately, there was no quantitative measure of method reproducibility in these previous studies. One advantage that comminution methods show over the RIAC, is that they typically produce larger suspension volumes. Kakran *et al.*^56^ demonstrated production of 40 mL (HPH), 20–50 mL (cavi-precipitation) and 150 mL (bead milling method). The drug concentration used in these methods is also greater, with quercetin at an initial concentration of 5–10% w/w. Notably, in a subsequent study, Kakran *et al.* demonstrated that the nanosuspensions produced with HPH and bead milling were stable both at 4 °C and room temperature for at least 180 days, whereas the nanosuspension produced with cavi-precipitation formed large aggregates that subsequently sedimented.^101^ They attributed this limited stability to the presence of organic solvent (DMSO or ethanol) in the formulation. A similar effect of the solvent is hypothesized to impact the formulation used in this work. More recently, Lucida *et al.* tested the production of quercetin nanocrystals utilising a planetary miller, in the presence of zirconium oxide milling beads.^59^ The method yielded quercetin nanocrystals in 30 minutes, with a mean particle size of 289.9 nm and PDI of 0.308. The obtained nanoparticles retained their dimension for up to 60 days at room temperature, however no data on the method reproducibility was reported in the study. As for other comminution methods, a greater initial quercetin concentration (10% w/w) was used compared to nanoprecipitation-based methods. Manca *et al.* produced quercetin nanocrystals with high reproducibility using a small-scale wet beads milling method, with 40 minutes of processing time.^98^ Quercetin was present at concentrations of 3% or 5% w/w, and two polymeric stabilizers were evaluated (KP188 and Tween 80, TW80) at a 1% w/v concentration. The obtained nanoparticles had a mean diameter and PDI of 326 ± 24 nm and 0.30 ± 0.02 (with 3% quercetin and 1% KP188) and of 431 ± 15 nm and 0.33 ± 0.01 (with 3% quercetin and 1% TW80). The obtained nanoparticles did not undergo size changes over 90 days at room temperature when TW80 was used. Nanoparticles produced in the presence of KP188 underwent a 27% size increase after 1 day, but then maintained their size for the remaining 89 days. As for previous studies using comminution methods, the produced particles possessed high stability. The size and size dispersity of particles obtained with this manufacturing approach were however greater than the ones obtained using the RIAC. In a following work, Kakran *et al.*^57^ evaluated the production of quercetin nanocrystals using a batch solvent-antisolvent method. They tested the effect of parameters such as quercetin concentration, solvent/antisolvent ratio, quercetin solution injection flow rate, and stirring speed of the antisolvent on nanocrystal size. Drug concentration was similar to that used in the present work; between 5 and 15 mg mL^−1^ in ethanol, but the antisolvent used was DI water only (*i.e.*, no stabilizer was employed). With this method they were able to produce relatively fine quercetin nanoparticles (between 170 nm and 560 nm in diameter). A quantitative measure of particle size dispersity, sample stability and experimental reproducibility was not included in this previous study. The processing time and final quercetin concentration were comparable to those achieved with the RIAC, although the RIAC produced quercetin nanocrystals that are generally smaller and with a narrower size distribution. To the best of authors' knowledge, no previous work focused on the microfluidic-based production of quercetin nanocrystals. Therefore, a direct comparison between the RIAC and a conventional microfluidic approach is not possible at the current time and could form the basis for future research.

Compared to other microfluidic-based nanoprecipitation techniques, such as the one used to manufacture hydrocortisone nanocrystals by Ali *et al.*,^84^ it is clear that one advantage of the RIAC is its simplicity of operation. In the cited research, drug nanoparticles were produced through a series of steps, comprising initial precipitation of the API in a microfluidic Y-shaped mixer, followed by batch agitation and sonication of the final suspension. Two micro-pumps were also required to convey the hydrocortisone solution and antisolvent through the reactor. The lack of such instrumentation is one of the initial barriers that some laboratories may encounter on their first venture into using microfluidic-based techniques. A second advantage of the RIAC is its ease of manufacture. The fabrication of microfluidic reactors can be relatively complex, costly, and dependent on multiple steps, as shown in the work by Arzi *et al.*^61^ RIAC manufacturing instead does not require any post-processing step, as the reactor is ready to use right after 3D printing. This allows for simpler and more cost-effective optimization of the device during development, as well as reproducible and frequent device substitution when required.

Despite the benefits of the developed production method, the quercetin nanosuspensions produced in this study however possessed limited stability. Even though suspension stabilization was not the primary focus of this work, preliminary attempts were carried out to address this issue. This would substantially form the basis for future work, which aims to specifically address nanosuspension stability. Upon production, nanocrystals were small in size and had a narrow size distribution, however after 4 to 12 hours larger particles formed that precipitated irreversibly. This could be due to either aggregation and/or Ostwald ripening.^25,102,103^ Storing samples at 4 °C reduced sample stability further, suggesting that particle aggregation may be the main contributing factor to the process. Many factors can affect stability of API nanoparticle suspensions; among these, three specific formulation aspects were identified that could potentially be improved to increase sample half-life. (1) It is possible that mixing within the RIACs is sufficiently effective to yield rapid nucleation and crystal precipitation, but the residence time is too short to allow complete surface coating by the stabilizer. (2) The presence of 10% ethanol in the final formulation (0.5 mL ethanol in 5 mL suspension) could potentially promote quercetin solubilization and Ostwald ripening, which could enhance particle growth over time.^102^ And (3) the ethanol present in the formulation could interact with the stabilizer, causing dehydration of the coating layer and reducing its ability to impair aggregation.^92^

**Fig. 11:**
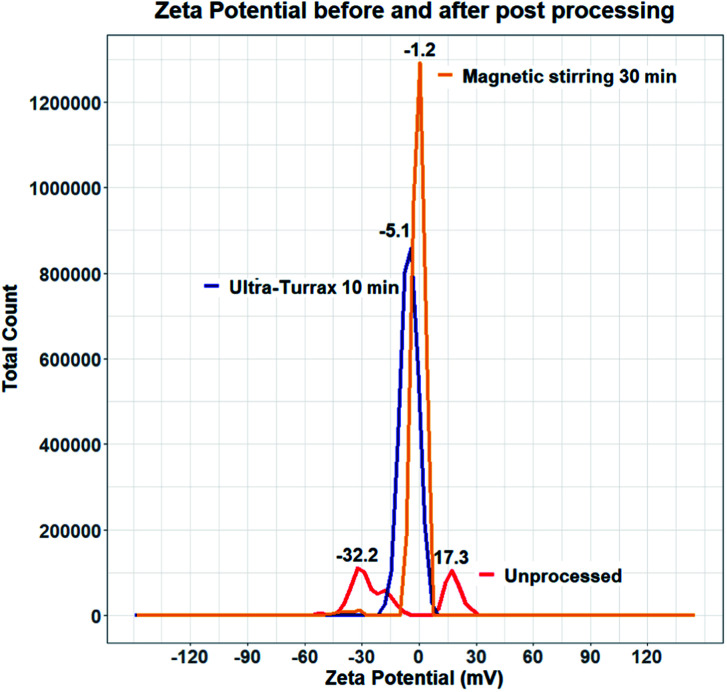
Zeta potential distribution of unprocessed and post-processed quercetin nanocrystal suspensions. Before processing the zeta potential distribution displays two separate peaks (red line), likely due to the presence of uncoated quercetin nanocrystals and negative polymeric micelles. After processing (orange and blue lines), sample zeta potential shifted and the distribution presents only one (slightly negative) peak.

Experiments were therefore carried out to determine whether the surface of quercetin nanocrystals was effectively coated by the polymeric stabilizer. Zeta potential measurements were performed on samples produced using HPMC 0.5% and KP407 1%. Data from the literature suggest that quercetin nanocrystals coated by KP407, should have a zeta potential of around −25 mV.^99^ The zeta potential distribution instead presented two separate peaks, at negative (−32.2 mV) and positive (+17.3 mV) values, respectively (Fig. 11). These peaks are likely due to the presence of negatively charged polymeric micelles and positively charged quercetin nanocrystals (either naked or partly coated). To enhance surface coating of quercetin nanoparticles, a post-processing step was introduced to favour stabilizer interaction with the nanoparticle surface. Two different approaches were attempted to achieve this: (i) stirring at 800 rpm for 30 minutes (using a magnetic stirrer), and (ii) homogenization for 10 minutes using the UltraTurrax homogenizer. Both methods yielded encouraging results, as shown in Fig. 11. After post-processing, the zeta potential profile displays a single, negative peak for both methods (at −5.176 mV after UltraTurrax homogenization, and −1.257 mV after magnetic stirring). Nanocrystal size after post-processing was also unaffected in both cases. Since KP407 was used as stabilizer in these experiments a more negative zeta potential was however expected, consistently with data from the literature.^99^ Unfortunately, even after post-processing, both samples still underwent rapid and irreversible sedimentation. Following this first attempt, removal of ethanol by centrifugation was subsequently evaluated. However, centrifuging the nanoparticle suspension for 150 minutes at 5000 RCF (the maximum speed allowed by the centrifuge available), did not result in effective particle sedimentation and separation from the supernatant. It is worth mentioning that freeze drying was also carried out as a potential alternative approach for the removal of ethanol. This resulted in the formation of large aggregates (data not shown) that did not return to the original nanoparticle size upon resuspension. The attempts to improve stability of the quercetin nanocrystal suspensions were unsuccessful. Future work should thus aim to investigate and address this challenge more comprehensively. In particular, data from the literature suggest that the presence of organic solvent in the formulation, is detrimental to quercetin nanocrystals stability.^57,101^ For this reason, future work should focus on optimising the RIAC design and particle production method to reduce the ethanol content in the end-product.

## Conclusions

This study demonstrated the potential of centrifugal flow-through reactors (also known as reactors-in-a-centrifuge, or RIACs) as a cost-effective, facile and pump-free technology for producing pharmaceutically relevant nanoparticulate systems. RIACs can be manufactured using a desktop 3D printer that does not require any post-manufacturing treatment before usage. They can be actuated using conventional laboratory centrifuges rather than specialist and costly pumps, making them suitable for widespread adoption. Users with expertise in technical drawing and rapid prototyping could customize the RIAC design to fit specific needs, as well as incorporate additional functionalities. To facilitate this, technical drawings of the reactors are provided as ESI.†

In this work, two RIAC architectures were developed, featuring a spiral shaped or straight mixing channel. These configurations are comparable to those commonly employed in microfluidic devices, currently used for nanoparticle production. The channel architectures were 3D printed within a cylindrical structure, which can be easily primed and placed within a centrifuge rotor. Both RIACs were capable of producing quercetin nanocrystals, with a therapeutically relevant diameter (between 190.60 ± 9.73 nm and 302.27 ± 33.154 nm). This was similar or smaller than particles obtained through precipitation and high energy methods in other works.^56,58,59,98,99,101^

The effects of changing production and formulation-related parameters on nanocrystal size were also demonstrated. Specifically, a significant increase in nanocrystal diameter was observed when the amount of viscosity enhancer (HPMC) was increased. This viscosity-dependent increase in particle size was more noticeable for samples prepared using the spiral-RIAC compared to the straight-RIAC. In addition, significant variations in nanocrystal diameter were detected when the manufacturing was carried out using different particle stabilizers. Varying the centrifugal force had a less pronounced effect on nanocrystal size, but interestingly this effect depended on the type of RIAC geometry used. Notably, within the parametric space investigated, most samples had a low size dispersity, with PDI values between 0.029 ± 0.008 and 0.100 ± 0.045 which are not easily achievable with other low-energy production methods. To the best of authors' knowledge, this is the first attempt at producing API nanocrystals using a 3D printed flow-through reactor solely actuated by a conventional laboratory centrifuge. Although it was not an original objective of the study, it was also demonstrated that under certain formulation conditions, RIACs can be utilised as a strategy for rapidly producing micellar systems with low size dispersity.

Quercetin was employed as a model drug in this research for its bioactive properties and intrinsic fluorescence. The method proposed here could be employed by researchers to formulate nanocrystals, which can be used as a model API nanocrystal for *in vitro* and *in vivo* research. Given the promising results obtained, it is anticipated that RIACs could also be employed to produce nanocrystal forms of other APIs in future research.

Importantly, future work should address the stability issues that were encountered with the current formulation and perform a more extensive characterization of the physical form of the obtained nanoparticles. Improved nanoparticle stability could also enable further characterisation of nanoparticle morphology, *i.e.* through transmission electron microscopy (TEM) imaging. In addition, a quantitative characterization of the flow dynamics and mixing regimes in the RIACs could be performed, to obtain an accurate estimate of residence time within the device. Computational fluid dynamics (CFD) models could contribute to further our understanding of the mixing performance of these reactors, allowing for design modifications that could improve RIACs production efficiency. Particle size measurements could also be performed at different time points of the production process, to potentially gain further information about the nanoparticle formation process.

Finally, future research could investigate scaling-up strategies to achieve greater particle production rates. This may involve increasing the overall dimensions of the reactor or operating multiple reactors simultaneously. Towards this, additional experiments could be performed to assess the reproducibility of nanoparticle production across multiple RIACs replica.

## Author contributions

Davide De Grandi performed the investigation, data collection and formal analysis, methodology optimization, and paper writing. Alireza Meghdadi supported with training and data analysis. Gareth LuTheryn supported with training and paper revision. Dario Carugo conceptualised and supervised the project, and contributed to paper writing and revision.

## Conflicts of interest

There are no conflicts to declare.

## Supplementary Material

RA-012-D2RA02745C-s001

RA-012-D2RA02745C-s002

RA-012-D2RA02745C-s003

## References

[cit1] Amidon G. L., Lennernäs H., Shah V. P., Crison J. R. (1995). Pharm. Res..

[cit2] Lipinski C. A. (2000). J. Pharmacol. Toxicol. Methods.

[cit3] Tao J., Chow S. F., Zheng Y. (2019). Acta Pharm. Sin. B.

[cit4] Shekhawat P. B., Pokharkar V. B. (2017). Acta Pharm. Sin. B.

[cit5] Guimarães D., Cavaco-Paulo A., Nogueira E. (2021). Int. J. Pharm..

[cit6] Ghasemiyeh P., Mohammadi-Samani S. (2018). Res. Pharm. Sci..

[cit7] Jaiswal M., Dudhe R., Sharma P. K. (2015). 3 Biotech.

[cit8] Jain N. K., Gupta U. (2008). Expert Opin. Drug Metab. Toxicol..

[cit9] Ahmad M. Z., Akhter S., Jain G. K., Rahman M., Pathan S. A., Ahmad F. J., Khar R. K. (2010). Expert Opin. Drug Delivery.

[cit10] Begines B., Ortiz T., Pérez-Aranda M., Martínez G., Merinero M., Argüelles-Arias F., Alcudia A. (2020). Nanomaterials.

[cit11] Mohammad I. S., Hu H., Yin L., He W. (2019). Int. J. Pharm..

[cit12] Hu J., Johnston K. P., Williams III R. O. (2004). Drug Dev. Ind. Pharm..

[cit13] Chang T.-L., Zhan H., Liang D., Liang J. F. (2015). Front. Chem. Sci. Eng..

[cit14] Colombo M., Staufenbiel S., Rühl E., Bodmeier R. (2017). Int. J. Pharm..

[cit15] Romero G. B., Keck C. M., Müller R. H., Bou-Chacra N. A. (2016). Eur. J. Pharm. Biopharm..

[cit16] Lv F., Wang J., Chen H., Sui L., Feng L., Liu Z., Liu Y., Wei G., Lu W. (2021). J. Controlled Release.

[cit17] Costabile G., Provenzano R., Azzalin A., Scoffone V. C., Chiarelli L. R., Rondelli V., Grillo I., Zinn T., Lepioshkin A., Savina S., Miro A., Quaglia F., Makarov V., Coenye T., Brocca P., Riccardi G., Buroni S., Ungaro F. (2020). Nanomedicine.

[cit18] Tian Z., Mai Y., Meng T., Ma S., Gou G., Yang J. (2021). AAPS PharmSciTech.

[cit19] McGuckin M. B., Wang J., Ghanma R., Qin N., Palma S. D., Donnelly R. F., Paredes A. J. (2022). J. Controlled Release.

[cit20] Lu Y., Li Y., Wu W. (2016). Acta Pharm. Sin. B.

[cit21] Tierney T., Bodnár K., Rasmuson Å., Hudson S. (2017). Int. J. Pharm..

[cit22] Studart A. R., Amstad E., Gauckler L. J. (2007). Langmuir.

[cit23] Singh B. P., Menchavez R., Takai C., Fuji M., Takahashi M. (2005). J. Colloid Interface Sci..

[cit24] Van Eerdenbrugh B., Van den Mooter G., Augustijns P. (2008). Int. J. Pharm..

[cit25] Sinha B., Müller R. H., Möschwitzer J. P. (2013). Int. J. Pharm..

[cit26] Gujar K., Wairkar S. (2020). Phytomedicine.

[cit27] Salazar J., Müller R. H., Möschwitzer J. P. (2013). J. Pharm. Sci..

[cit28] Raghava Srivalli K. M., Mishra B. (2016). Saudi Pharm. J..

[cit29] Bhakay A., Rahman M., Dave R. N., Bilgili E. (2018). Pharmaceutics.

[cit30] Webb C., Forbes N., Roces C. B., Anderluzzi G., Lou G., Abraham S., Ingalls L., Marshall K., Leaver T. J., Watts J. A., Aylott J. W., Perrie Y. (2020). Int. J. Pharm..

[cit31] Khizar S., Zine N., Errachid A., Jaffrezic-Renault N., Elaissari A. (2022). Electrophoresis.

[cit32] Hamdallah S. I., Zoqlam R., Erfle P., Blyth M., Alkilany A. M., Dietzel A., Qi S. (2020). Int. J. Pharm..

[cit33] LaMer V. K., Dinegar R. H. (1950). J. Am. Chem. Soc..

[cit34] Thanh N. T. K., Maclean N., Mahiddine S. (2014). Chem. Rev..

[cit35] Valencia P. M., Farokhzad O. C., Karnik R., Langer R. (2012). Nat. Nanotechnol..

[cit36] Ding S., Anton N., Vandamme T. F., Serra C. A. (2016). Expert Opin. Drug Delivery.

[cit37] Shrimal P., Jadeja G., Patel S. (2020). Chem. Eng. Res. Des..

[cit38] Niculescu A.-G., Chircov C., Bîrcă A. C., Grumezescu A. M. (2021). Nanomaterials.

[cit39] Giorello A., Nicastro A., Berli C. L. A. (2022). Adv. Mater. Technol..

[cit40] Ma J., Ming-Yuen Lee S., Yi C., Li C.-W. (2017). Lab Chip.

[cit41] Jeong H.-H., Issadore D., Lee D. (2016). Korean J. Chem. Eng..

[cit42] Dressaire E., Sauret A. (2016). Soft Matter.

[cit43] Hoehl M. M., Schulte Bocholt E., Kloke A., Paust N., von Stetten F., Zengerle R., Steigert J., Slocum A. H. (2014). Analyst.

[cit44] Kloke A., Fiebach A. R., Zhang S., Drechsel L., Niekrawietz S., Hoehl M. M., Kneusel R., Panthel K., Steigert J., von Stetten F., Zengerle R., Paust N. (2014). Lab Chip.

[cit45] Kong L. X., Perebikovsky A., Moebius J., Kulinsky L., Madou M. (2016). J. Lab. Autom..

[cit46] Andrea Cristaldi D., Labanca A., Donal Pottinger T., Owen J., Stulz E., Zhang X., Carugo D. (2021). Chem. Eng. J..

[cit47] Volk A. A., Epps R. W., Abolhasani M. (2021). Adv. Mater..

[cit48] Paccotti N., Chiadò A., Novara C., Rivolo P., Montesi D., Geobaldo F., Giorgis F. (2021). Biosensors.

[cit49] Cai Q., Castagnola V., Boselli L., Moura A., Lopez H., Zhang W., de Araújo J. M., Dawson K. A. (2022). Nanoscale Horiz..

[cit50] Tian F., Cai L., Liu C., Sun J. (2022). Lab Chip.

[cit51] Shin Y., Lim Y., Kwak T., Hwang J. H., Georgescu A., Huh D., Kim D., Kang T. (2021). Adv. Funct. Mater..

[cit52] Erlund I. (2004). Nutr. Res..

[cit53] Li Y., Yao J., Han C., Yang J., Chaudhry M. T., Wang S., Liu H., Yin Y. (2016). Nutrients.

[cit54] Ulusoy H. G., Sanlier N. (2020). Crit. Rev. Food Sci. Nutr..

[cit55] Wang L., Song J., Liu A., Xiao B., Li S., Wen Z., Lu Y., Du G. (2020). Nat. Prod. Bioprospect..

[cit56] Kakran M., Shegokar R., Sahoo N. G., Al Shaal L., Li L., Müller R. H. (2012). Eur. J. Pharm. Biopharm..

[cit57] Kakran M., Sahoo N. G., Li L., Judeh Z. (2012). Powder Technol..

[cit58] Sahoo N. G., Kakran M., Shaal L. A., Li L., Müller R. H., Pal M., Tan L. P. (2011). J. Pharm. Sci..

[cit59] Lucida H., Febriyenti F., Pradana R., Rahmatika L. (2016). Der Pharm. Lett..

[cit60] Schweiger S., Jungbauer A. (2018). J. Chromatogr. A.

[cit61] Arzi R. S., Kay A., Raychman Y., Sosnik A. (2021). Pharmaceutics.

[cit62] Khan I. U., Serra C. A., Anton N., Vandamme T. F. (2015). Expert Opin. Drug Delivery.

[cit63] Odetade D. F., Vladisavljevic G. T. (2016). Micromachines.

[cit64] Ranjita S. (2013). J. Pharm. Invest..

[cit65] Junghanns J.-U. A. H., Müller R. H. (2008). Int. J. Nanomed..

[cit66] Attama A. A. , Reginald-Opara J. N., Uronnachi E. M. and Onuigbo E. B., in Nanoscience in Dermatology, ed. M. R. Hamblin, P. Avci and T. W. Prow, Academic Press, Boston, 2016, pp. 323–336

[cit67] Singh A. K. , Sharma A. K., Khan I., Gothwal A., Gupta L. and Gupta U., in Nanostructures for Oral Medicine, ed. E. Andronescu and A. M. Grumezescu, Elsevier, 2017, pp. 231–261

[cit68] Casanova A. G., Prieto M., Colino C. I., Gutiérrez-Millán C., Ruszkowska-Ciastek B., de Paz E., Martín Á., Morales A. I., López-Hernández F. J. (2021). Int. J. Mol. Sci..

[cit69] Lopes K., Cavalcante I., Silva R., Brito D., Fechine L., Moreira D., Vieira Í., Azu F., Leal L., Ribeiro M., Ricardo N. (2020). Quim. Nova.

[cit70] Tiwari S., Ma J., Rathod S., Bahadur P. (2021). Colloids Surf., A.

[cit71] Vekilov P. G. (2010). Cryst. Growth Des..

[cit72] Tuomela A., Hirvonen J., Peltonen L. (2016). Pharmaceutics.

[cit73] Zuo B., Sun Y., Li H., Liu X., Zhai Y., Sun J., He Z. (2013). Int. J. Pharm..

[cit74] Tuomela A., Liu P., Puranen J., Rönkkö S., Laaksonen T., Kalesnykas G., Oksala O., Ilkka J., Laru J., Järvinen K., Hirvonen J., Peltonen L. (2014). Int. J. Pharm..

[cit75] Ghosh I., Schenck D., Bose S., Liu F., Motto M. (2013). Pharm. Dev. Technol..

[cit76] Shi H., Zhao Y., Liu Z. (2020). Sens. Actuators, B.

[cit77] Thiele M., Knauer A., Malsch D., Csáki A., Henkel T., Köhler J. M., Fritzsche W. (2017). Lab Chip.

[cit78] van Oss C. J., Giese R. F., Costanzo P. M. (1990). Clays Clay Miner..

[cit79] Duran J. D. G., Guindo M. C., Delgado A. V., Gonzalez-Caballero F. (1995). Langmuir.

[cit80] Studart A. R., Amstad E., Gauckler L. J. (2007). Langmuir.

[cit81] Bandulasena M. V., Vladisavljević G. T., Odunmbaku O. G., Benyahia B. (2017). Chem. Eng. Sci..

[cit82] Chiesa E., Dorati R., Pisani S., Conti B., Bergamini G., Modena T., Genta I. (2018). Pharmaceutics.

[cit83] Chiesa E., Dorati R., Modena T., Conti B., Genta I. (2018). Int. J. Pharm..

[cit84] Ali H. S. M., York P., Blagden N. (2009). Int. J. Pharm..

[cit85] Lababidi N., Sigal V., Koenneke A., Schwarzkopf K., Manz A., Schneider M. (2019). Beilstein J. Nanotechnol..

[cit86] Gdowski A., Johnson K., Shah S., Gryczynski I., Vishwanatha J., Ranjan A. (2018). J. Nanobiotechnol..

[cit87] Dong Y., Ng W. K., Hu J., Shen S., Tan R. B. H. (2010). Int. J. Pharm..

[cit88] Gimondi S., Guimarães C. F., Vieira S. F., Gonçalves V. M. F., Tiritan M. E., Reis R. L., Ferreira H., Neves N. M. (2022). Nanomedicine.

[cit89] Horn D., Rieger J. (2001). Angew. Chem., Int. Ed..

[cit90] Bai S., Li W.-S., Liu W., Luo Y., Chu G.-W., Chen J.-F. (2022). Chem. Eng. Sci..

[cit91] Bodratti A. M., Alexandridis P. (2018). J. Funct. Biomater..

[cit92] Wu L., Zhang J., Watanabe W. (2011). Adv. Drug Delivery Rev..

[cit93] Rabinow B. E. (2004). Nat. Rev. Drug Discovery.

[cit94] Nutan M. T. H. and Reddy I. K., in Pharmaceutical Suspensions: From Formulation Development to Manufacturing, ed. A. K. Kulshreshtha, O. N. Singh and G. M. Wall, Springer, New York, NY, 2010, pp. 39–65

[cit95] Choi J.-Y., Yoo J. Y., Kwak H.-S., Uk Nam B., Lee J. (2005). Curr. Appl. Phys..

[cit96] Lee J., Choi J.-Y., Park C. H. (2008). Int. J. Pharm..

[cit97] Eckert R. W. , Hartmann S. F. and Keck C. M., Small-scale bead milling process characterization: an approach towards tailor-made nanocrystals, European Conference on Pharmaceutics, Bologna, Italy, 2019

[cit98] Manca M. L., Lai F., Pireddu R., Valenti D., Schlich M., Pini E., Ailuno G., Fadda A. M., Sinico C. (2020). J. Drug Delivery Sci. Technol..

[cit99] Hatahet T., Morille M., Hommoss A., Dorandeu C., Müller R. H., Bégu S. (2016). Eur. J. Pharm. Biopharm..

[cit100] Alshora D. H., Ibrahim M. A., Elzayat E., Almeanazel O. T., Alanazi F. (2018). PLoS One.

[cit101] Kakran M., Shegokar R., Sahoo N. G., Gohla S., Li L., Müller R. H. (2012). J. Pharm. Pharmacol..

[cit102] Verma S., Kumar S., Gokhale R., Burgess D. J. (2011). Int. J. Pharm..

[cit103] Wang Y., Zheng Y., Zhang L., Wang Q., Zhang D. (2013). J. Controlled Release.

