# Supplementary material for: Facile production of quercetin nanoparticles using 3D printed centrifugal flow reactors
Source: RSC Adv. 2022 Jul 19;12(32):20696–713. doi: 10.1039/d2ra02745c (PMC9295137; doi:10.1039/d2ra02745c)
Supplement: RA-012-D2RA02745C-s003 [file RA-012-D2RA02745C-s003.pdf]

Electronic Supplementary Material (ESI) for RSC Advances.
This journal is © The Royal Society of Chemistry 2022

Supplementary Material for:

Facile production of quercetin nanoparticles using 3D printed centrifugal flow reactors

Davide De Grandi, [a,b] Alireza Meghdadi, [b,c] Gareth LuTheryn [b] and Dario Carugo [†,b]

[a] Department of Drug Sciences, Faculty of Pharmacy, University of Pavia, Pavia 27100, Italy.
[b] Department of Pharmaceutics, School of Pharmacy, University College London, London WC1N 1AX, United Kingdom
[c] Department of Mechanical Engineering, Faculty of Engineering and Physical Sciences, University of Southampton, Southampton SO17 1BJ, United Kingdom.
† Article correspondence to: Dr Dario Carugo, d.carugo@ucl.ac.uk

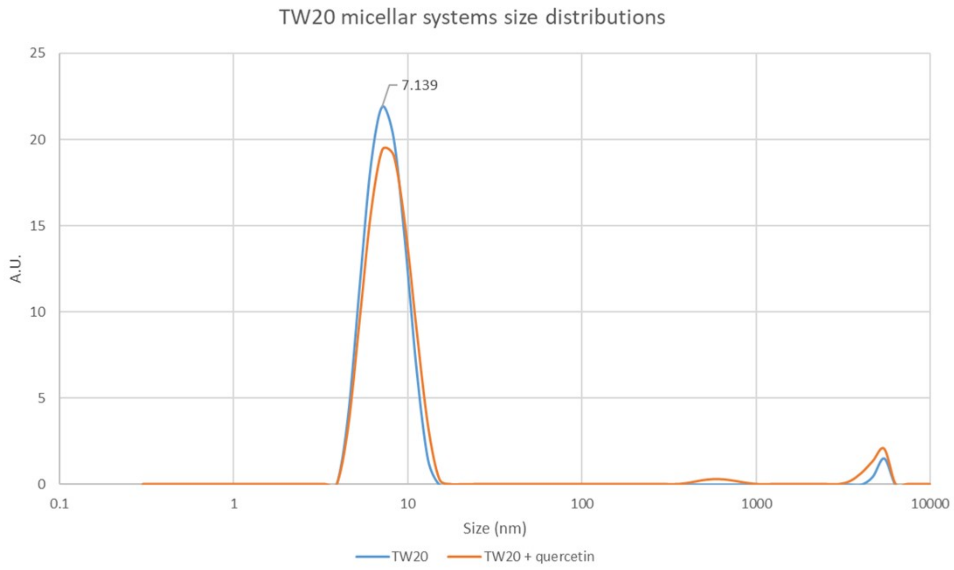

Figure S1 Size distribution of a micellar dispersion of TW20 4% in the absence (blue) and presence (orange) of quercetin. Micelles show a comparable dimension both in the presence or absence of quercetin.

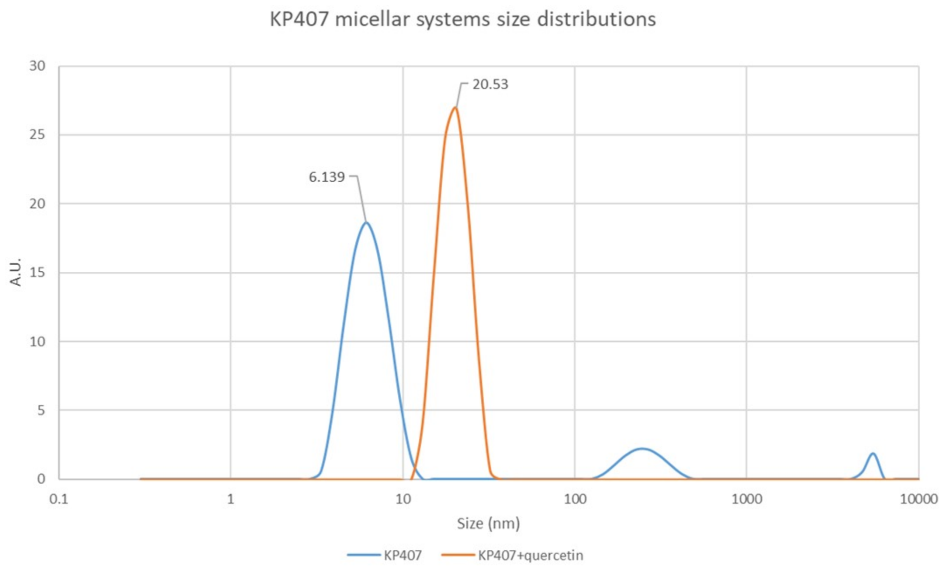

Figure S2 – Size distribution of a micellar dispersion of KP407 4% in the absence (blue) and presence (orange) of quercetin. There is a substantial size difference between the two samples, which may be attributed to the presence of quercetin molecules in the micellar structure.

Table S1 – Data frame of samples manufactured using increasing HPMC concentration (0.5%, 0.75%, 1%).

Sample	RCF	Focal_Var	RIAC	Size	PDI
500_H.05_K4.1_SP	500RCF	HPMC_05	SPIRAL	196.7	0.09633
500_H.05_K4.1_SP	500RCF	HPMC_05	SPIRAL	196.1	0.069695
500_H.05_K4.1_SP	500RCF	HPMC_05	SPIRAL	201.2	0.025106
500_H.05_K4.1_ST	500RCF	HPMC_05	STRAIGHT	226.8	0.086568
500_H.05_K4.1_ST	500RCF	HPMC_05	STRAIGHT	255.6	0.141242
500_H.05_K4.1_ST	500RCF	HPMC_05	STRAIGHT	244.6	0.052753
3000_H.05_K4.1_SP	3000RCF	HPMC_05	SPIRAL	181.3	0.032571
3000_H.05_K4.1_SP	3000RCF	HPMC_05	SPIRAL	200.7	0.09402
3000_H.05_K4.1_SP	3000RCF	HPMC_05	SPIRAL	189.8	0.085259
3000_H.05_K4.1_ST	3000RCF	HPMC_05	STRAIGHT	234.2	0.128214
3000_H.05_K4.1_ST	3000RCF	HPMC_05	STRAIGHT	245.4	0.034166
3000_H.05_K4.1_ST	3000RCF	HPMC_05	STRAIGHT	225.9	0.060252
500_H.075_K4.1_SP	500RCF	HPMC_075	SPIRAL	255.8	0.055036
500_H.075_K4.1_SP	500RCF	HPMC_075	SPIRAL	249.5	0.097609
500_H.075_K4.1_SP	500RCF	HPMC_075	SPIRAL	248.3	0.055129
500_H.075_K4.1_ST	500RCF	HPMC_075	STRAIGHT	248.8	0.047878
500_H.075_K4.1_ST	500RCF	HPMC_075	STRAIGHT	272.2	0.131559
500_H.075_K4.1_ST	500RCF	HPMC_075	STRAIGHT	259.9	0.094298
3000_H.075_K4.1_SP	3000RCF	HPMC_075	SPIRAL	215.9	0.049779
3000_H.075_K4.1_SP	3000RCF	HPMC_075	SPIRAL	300.1	0.133867
3000_H.075_K4.1_SP	3000RCF	HPMC_075	SPIRAL	230.3	0.11767
3000_H.075_K4.1_ST	3000RCF	HPMC_075	STRAIGHT	207.3	0.042667
3000_H.075_K4.1_ST	3000RCF	HPMC_075	STRAIGHT	215.9	0.058861
3000_H.075_K4.1_ST	3000RCF	HPMC_075	STRAIGHT	247.3	0.100299
500_H.1_K4.1_SP	500RCF	HPMC_1	SPIRAL	229.7	0.020954
500_H.1_K4.1_SP	500RCF	HPMC_1	SPIRAL	310.7	0.03005
500_H.1_K4.1_SP	500RCF	HPMC_1	SPIRAL	280.2	0.036511
500_H.1_K4.1_ST	500RCF	HPMC_1	STRAIGHT	243.5	0.036295
500_H.1_K4.1_ST	500RCF	HPMC_1	STRAIGHT	246.9	0.108961
500_H.1_K4.1_ST	500RCF	HPMC_1	STRAIGHT	296	0.060489
3000_H.1_K4.1_SP	3000RCF	HPMC_1	SPIRAL	209.1	0.027128
3000_H.1_K4.1_SP	3000RCF	HPMC_1	SPIRAL	264.2	0.016338
3000_H.1_K4.1_SP	3000RCF	HPMC_1	SPIRAL	356.4	0.127981
3000_H.1_K4.1_ST	3000RCF	HPMC_1	STRAIGHT	267.7	0.038696
3000_H.1_K4.1_ST	3000RCF	HPMC_1	STRAIGHT	305.3	0.110747
3000_H.1_K4.1_ST	3000RCF	HPMC_1	STRAIGHT	333.8	0.142711

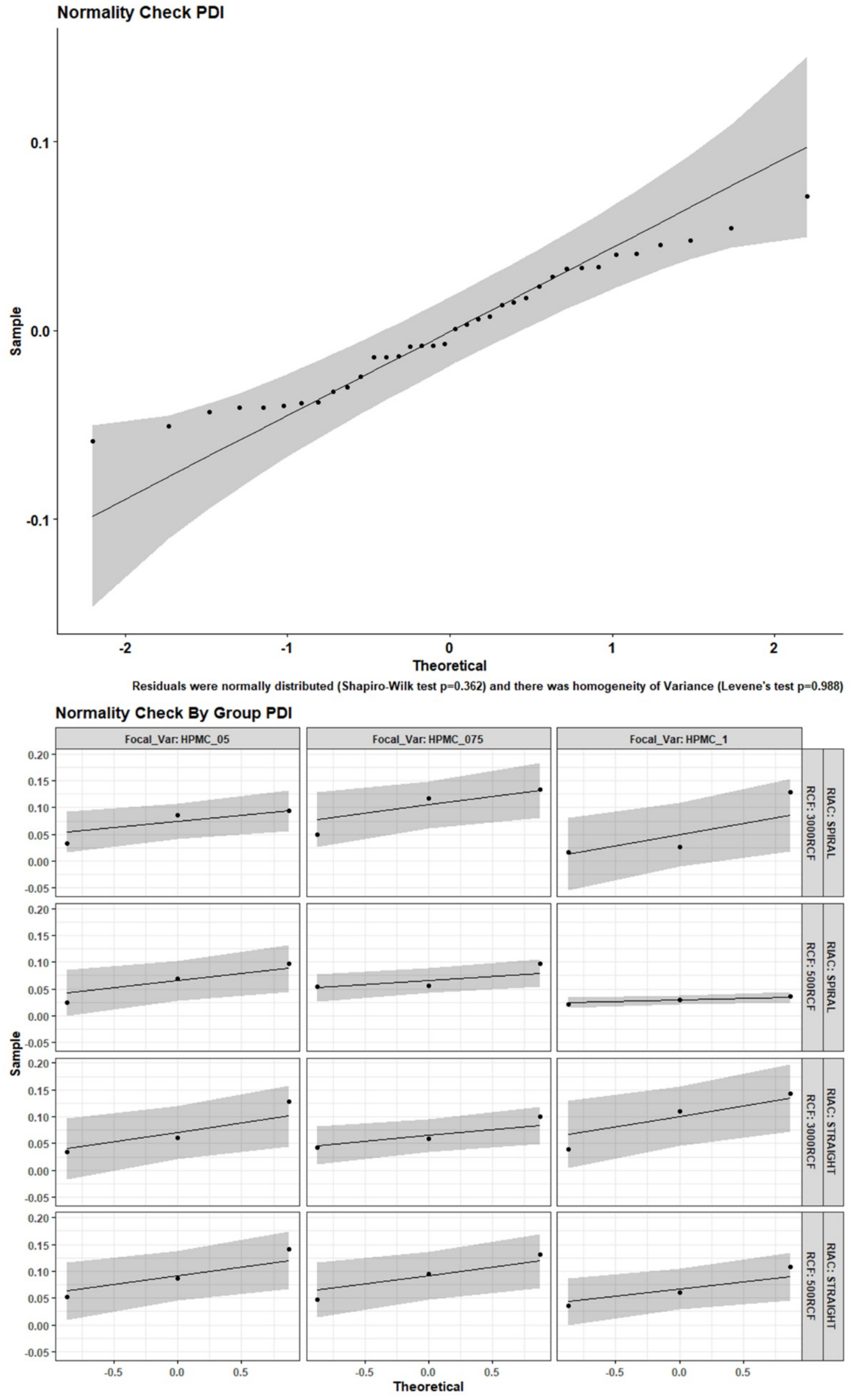

Figure S3 – Evaluation of ANOVA assumptions. The residuals of the linear model computed using the PDI data in Table S1 were normally distributed, and the Homogeneity of Variance assumption is also met. Shapiro-Wilk and Levene's tests were run to prove the validity of the assumptions.

Table S2 – Results of Three-Way ANOVA carried out to evaluate the effect of different independent variables on the PDI.

Three-Way ANOVA PDI						
Effect	DFn	DFd	F	p	p<.05	ges
RIAC	1	24	1.553	0.225		0.061
RCF	1	24	0.398	0.534		0.016
Focal_Var	2	24	0.668	0.522		0.053
RIAC:RCF	1	24	0.971	0.334		0.039
RIAC:Focal_Var	2	24	0.926	0.41		0.072
RCF:Focal_Var	2	24	0.571	0.573		0.045
RIAC:RCF:Focal_Var	2	24	0.352	0.707		0.028

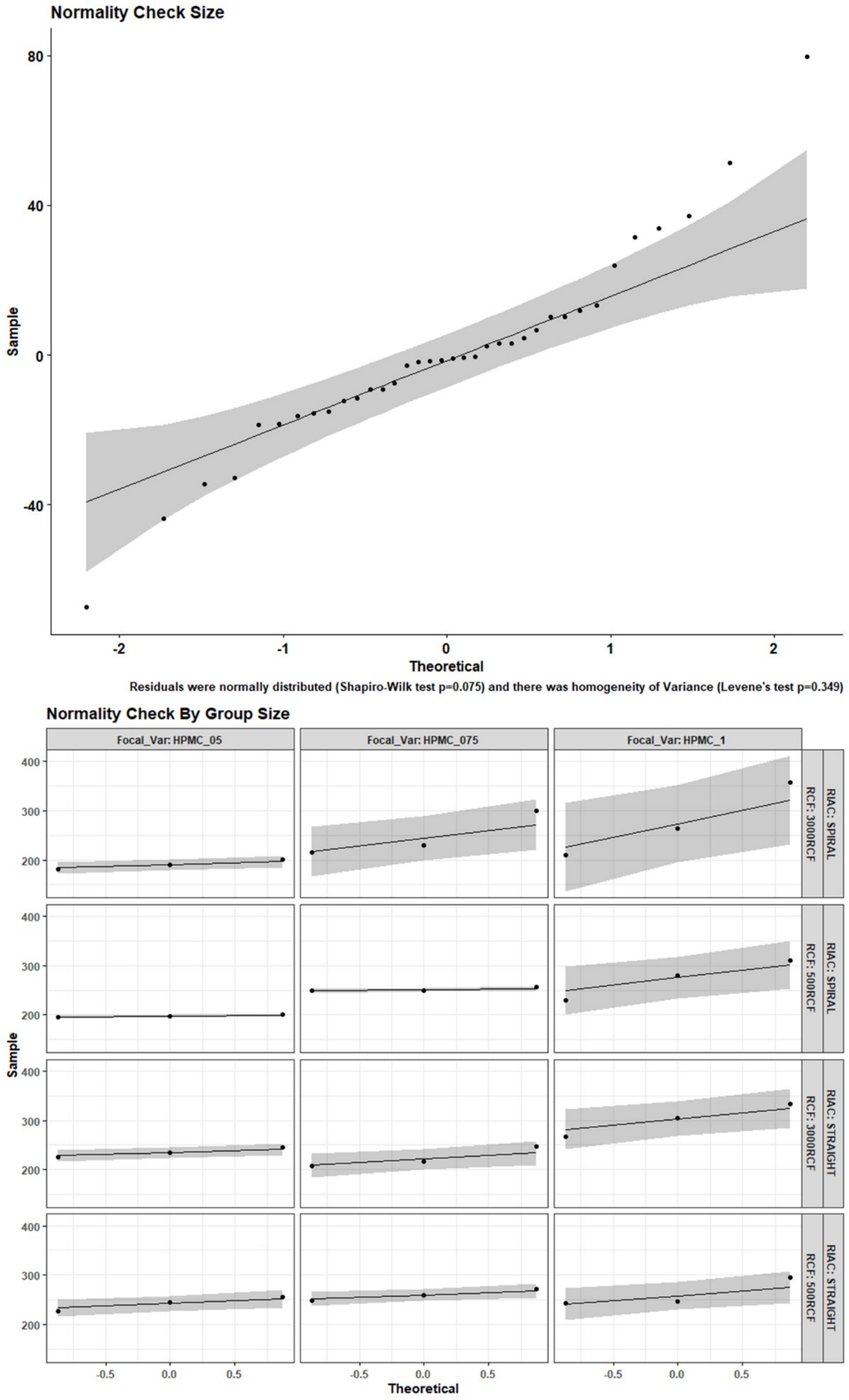

Figure S4 - Evaluation of ANOVA assumptions. The residuals of the linear model computed using the particle size data in Table S1 were normally distributed, and the Homogeneity of Variance assumption is also met. Shapiro-Wilk and Levene's tests were run to prove the validity of the assumptions.

Table S3 - Results of Three-Way ANOVA carried out to evaluate the effect of different independent variables on nanoparticle mean diameter.

Three-Way ANOVA Size						
Effect	DFn	DFd	F	p	p<.05	ges
RIAC	1	24	1.861	0.185		0.072
RCF	1	24	0.028	0.869		0.001
Focal_Var	2	24	11.382	3.34E-04	*	0.487
RIAC:RCF	1	24	0.002	0.963		9.01E-05
RIAC:Focal_Var	2	24	2.154	0.138		0.152
RCF:Focal_Var	2	24	1.318	0.286		0.099
RIAC:RCF:Focal_Var	2	24	0.942	0.404		0.073

Table S4 – Pairwise comparisons (t-test, Bonferroni adjusted). Nanocrystal size of samples grouped in four different ways was compared to identify any statistically significant difference.

Pairwise comparison_1 (t-test, Bonferroni adjust) Size															
RCF	Focal_Var	.y.	group1	group2	n1	n2	p	p.signif	p.adj	p.adj.signif	y.position	groups	x	xmin	xmax
3000RCF	HPMC_05	Size	SPIRAL	STRAIGHT	3	3	0.00501	**	0.00501	**	264.084		1	0.8	1.2
3000RCF	HPMC_075	Size	SPIRAL	STRAIGHT	3	3	0.428	ns	0.428	ns	318.784		2	1.8	2.2
3000RCF	HPMC_1	Size	SPIRAL	STRAIGHT	3	3	0.614	ns	0.614	ns	375.084		3	2.8	3.2
500RCF	HPMC_05	Size	SPIRAL	STRAIGHT	3	3	0.00657	**	0.00657	**	274.284		1	0.8	1.2
500RCF	HPMC_075	Size	SPIRAL	STRAIGHT	3	3	0.272	ns	0.272	ns	290.884		2	1.8	2.2
500RCF	HPMC_1	Size	SPIRAL	STRAIGHT	3	3	0.715	ns	0.715	ns	329.384		3	2.8	3.2

Pairwise comparison_2 (t-test, Bonferroni adjust) Size															
Focal_Var	RIAC	.y.	group1	group2	n1	n2	p	p.signif	p.adj	p.adj.signif	y.position	groups	x	xmin	xmax
HPMC_05	SPIRAL	Size	3000RCF	500RCF	3	3	0.274	ns	0.274	ns	219.884		1	0.8	1.2
HPMC_075	SPIRAL	Size	3000RCF	500RCF	3	3	0.93	ns	0.93	ns	318.784		2	1.8	2.2
HPMC_1	SPIRAL	Size	3000RCF	500RCF	3	3	0.954	ns	0.954	ns	375.084		3	2.8	3.2
HPMC_05	STRAIGHT	Size	3000RCF	500RCF	3	3	0.518	ns	0.518	ns	274.284		1	0.8	1.2
HPMC_075	STRAIGHT	Size	3000RCF	500RCF	3	3	0.0572	ns	0.0572	ns	290.884		2	1.8	2.2
HPMC_1	STRAIGHT	Size	3000RCF	500RCF	3	3	0.192	ns	0.192	ns	352.484		3	2.8	3.2

Pairwise comparison_3 (t-test, Bonferroni adjust) Size														
RCF	RIAC	.y.	group1	group2	n1	n2	p	p.signif	p.adj	p.adj.signif	y.position	groups	xmin	xmax
3000RCF	SPIRAL	Size	HPMC_05	HPMC_075	3	3	0.208	ns	0.625	ns	375.084		1	2

3000RCF	SPIRAL	Size	HPMC_05	HPMC_1	3	3	0.0823	ns	0.247	ns	403.11		1	3
3000RCF	SPIRAL	Size	HPMC_075	HPMC_1	3	3	0.526	ns	1	ns	431.136		2	3
500RCF	SPIRAL	Size	HPMC_05	HPMC_075	3	3	0.0338	*	0.101	ns	329.384		1	2
500RCF	SPIRAL	Size	HPMC_05	HPMC_1	3	3	0.00809	**	0.0243	*	357.41		1	3
500RCF	SPIRAL	Size	HPMC_075	HPMC_1	3	3	0.294	ns	0.882	ns	385.436		2	3
3000RCF	STRAIGHT	Size	HPMC_05	HPMC_075	3	3	0.563	ns	1	ns	352.484		1	2
3000RCF	STRAIGHT	Size	HPMC_05	HPMC_1	3	3	0.0126	*	0.0377	*	380.51		1	3
3000RCF	STRAIGHT	Size	HPMC_075	HPMC_1	3	3	0.00616	**	0.0185	*	408.536		2	3
500RCF	STRAIGHT	Size	HPMC_05	HPMC_075	3	3	0.315	ns	0.946	ns	314.684		1	2
500RCF	STRAIGHT	Size	HPMC_05	HPMC_1	3	3	0.273	ns	0.819	ns	342.71		1	3
500RCF	STRAIGHT	Size	HPMC_075	HPMC_1	3	3	0.915	ns	1	ns	370.736		2	3

Pairwise comparison_4 (t-test, Bonferroni adjust) Size													
RIAC	.y.	group1	group2	n1	n2	p	p.signif	p.adj	p.adj.signif	y.position	groups	xmin	xmax
SPIRAL	Size	HPMC_05	HPMC_075	6	6	0.0157	*	0.0472	*	375.024		1	2
SPIRAL	Size	HPMC_05	HPMC_1	6	6	0.00129	**	0.00386	**	402.96		1	3
SPIRAL	Size	HPMC_075	HPMC_1	6	6	0.239	ns	0.718	ns	430.896		2	3
STRAIGHT	Size	HPMC_05	HPMC_075	6	6	0.837	ns	1	ns	352.424		1	2
STRAIGHT	Size	HPMC_05	HPMC_1	6	6	0.0114	*	0.0342	*	380.36		1	3
STRAIGHT	Size	HPMC_075	HPMC_1	6	6	0.0174	*	0.0521	ns	408.296		2	3

Table S5 – Data frame of samples manufactured using different stabilizers (KP188, KP407, TW20).

Sample	RCF	Focal_Var	RIAC	Size	PDI
500_H.05_K18.1_SP	500RCF	KP188	SPIRAL	221.4	0.030255
500_H.05_K18.1_SP	500RCF	KP188	SPIRAL	230.2	0.109084
500_H.05_K18.1_SP	500RCF	KP188	SPIRAL	250.1	0.083246
500_H.05_K18.1_ST	500RCF	KP188	STRAIGHT	237.1	0.068004
500_H.05_K18.1_ST	500RCF	KP188	STRAIGHT	245.4	0.094906
500_H.05_K18.1_ST	500RCF	KP188	STRAIGHT	228.7	0.109678
3000_H.05_K18.1_SP	3000RCF	KP188	SPIRAL	238	0.089572
3000_H.05_K18.1_SP	3000RCF	KP188	SPIRAL	232.4	0.125653
3000_H.05_K18.1_SP	3000RCF	KP188	SPIRAL	248.2	0.111855
3000_H.05_K18.1_ST	3000RCF	KP188	STRAIGHT	257.2	0.06675
3000_H.05_K18.1_ST	3000RCF	KP188	STRAIGHT	249.5	0.094258
3000_H.05_K18.1_ST	3000RCF	KP188	STRAIGHT	263.1	0.142391
500_H.05_K4.1_SP	500RCF	KP407	SPIRAL	196.7	0.09633
500_H.05_K4.1_SP	500RCF	KP407	SPIRAL	196.1	0.069695
500_H.05_K4.1_SP	500RCF	KP407	SPIRAL	201.2	0.025106
500_H.05_K4.1_ST	500RCF	KP407	STRAIGHT	226.8	0.086568
500_H.05_K4.1_ST	500RCF	KP407	STRAIGHT	255.6	0.141242
500_H.05_K4.1_ST	500RCF	KP407	STRAIGHT	244.6	0.052753
3000_H.05_K4.1_SP	3000RCF	KP407	SPIRAL	181.3	0.032571
3000_H.05_K4.1_SP	3000RCF	KP407	SPIRAL	200.7	0.09402
3000_H.05_K4.1_SP	3000RCF	KP407	SPIRAL	189.8	0.085259
3000_H.05_K4.1_ST	3000RCF	KP407	STRAIGHT	234.2	0.128214
3000_H.05_K4.1_ST	3000RCF	KP407	STRAIGHT	245.4	0.034166
3000_H.05_K4.1_ST	3000RCF	KP407	STRAIGHT	225.9	0.060252
500_H.05_TW2.1_SP	500RCF	TW20	SPIRAL	220.9	0.057175
500_H.05_TW2.1_SP	500RCF	TW20	SPIRAL	225.3	0.073691
500_H.05_TW2.1_SP	500RCF	TW20	SPIRAL	221.5	0.120439
500_H.05_TW2.1_ST	500RCF	TW20	STRAIGHT	216.2	0.098663
500_H.05_TW2.1_ST	500RCF	TW20	STRAIGHT	239	0.086618
500_H.05_TW2.1_ST	500RCF	TW20	STRAIGHT	228.6	0.070622
3000_H.05_TW2.1_SP	3000RCF	TW20	SPIRAL	218.8	0.06713
3000_H.05_TW2.1_SP	3000RCF	TW20	SPIRAL	212.1	0.060269
3000_H.05_TW2.1_SP	3000RCF	TW20	SPIRAL	206.9	0.081428
3000_H.05_TW2.1_ST	3000RCF	TW20	STRAIGHT	206.4	0.102437
3000_H.05_TW2.1_ST	3000RCF	TW20	STRAIGHT	227.9	0.060898
3000_H.05_TW2.1_ST	3000RCF	TW20	STRAIGHT	202.8	0.056746

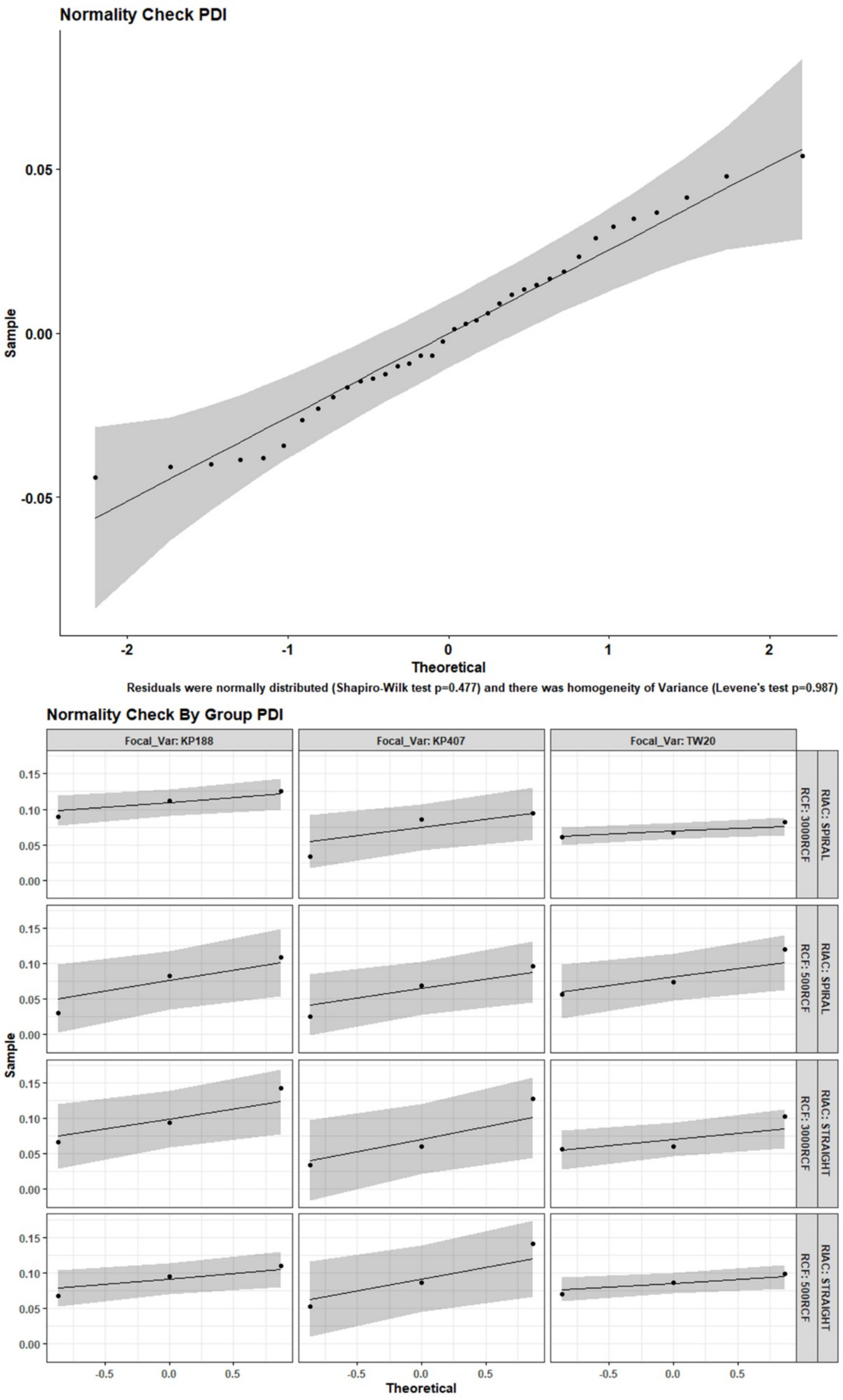

Figure S5 - Evaluation of ANOVA assumptions. The residuals of the linear model computed using the PDI data in Table S2 were normally distributed, and the Homogeneity of Variance assumption is also met. Shapiro-Wilk and Levene's tests were run to prove the validity of the assumptions.

Table S6 - Results of Three-Way ANOVA carried out to evaluate the effect of different independent variables on the PDI.

Three-Way ANOVA PDI						
Effect	DFn	DFd	F	p	p<.05	ges
RIAC	1	24	0.535	0		0.022
RCF	1	24	0.01	1		4.30E-04
Focal_Var	2	24	1.121	0		0.085
RIAC:RCF	1	24	0.56	0		0.023
RIAC:Focal_Var	2	24	0.167	1		0.014
RCF:Focal_Var	2	24	1.016	0		0.078
RIAC:RCF:Focal_Var	2	24	0.181	1		0.015

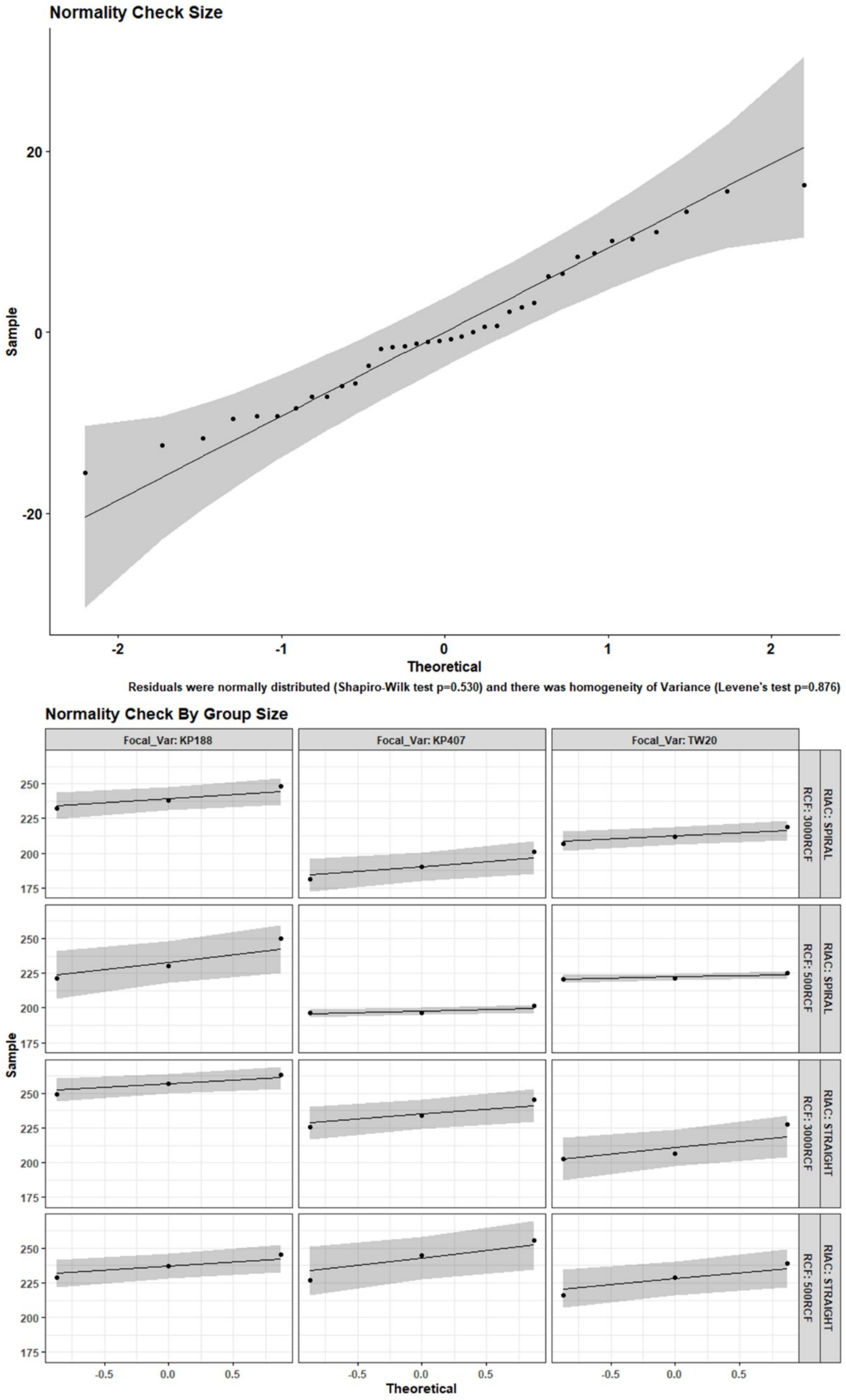

Figure S6 Evaluation of ANOVA assumptions. The residuals of the linear model computed using the particle size data in Table S2 were normally distributed, and the Homogeneity of Variance assumption is also met. Shapiro-Wilk and Levene's tests were run to prove the validity of the assumptions.

Table S7 - Results of Three-Way ANOVA carried out to evaluate the effect of different independent variables on nanoparticle mean diameter.

Three-Way ANOVA Size						
Effect	DFn	DFd	F	p	p<.05	ges
RIAC	1	24	33.719	5.49E-06	*	0.584
RCF	1	24	0.576	0.455		0.023
Focal_Var	2	24	24.127	1.80E-06	*	0.668
RIAC:RCF	1	24	0.188	0.668		8.00E-03
RIAC:Focal_Var	2	24	15.443	4.89E-05	*	0.563
RCF:Focal_Var	2	24	5.513	0.011	*	0.315

Table S8 – Pairwise comparisons (t-test, Bonferroni adjusted). Nanocrystal size of samples grouped in four different ways was compared to identify any statistically significant difference.

Pairwise comparison_1 (t-test, Bonferroni adjust) Size															
RCF	Focal_Var	.y.	group1	group2	n1	n2	p	p.signif	p.adj	p.adj.signif	y.position	groups	x	xmin	xmax
3000RCF	KP188	Size	SPIRAL	STRAIGHT	3	3	0.0483	*	0.0483	*	270.588		1	0.8	1.2
3000RCF	KP407	Size	SPIRAL	STRAIGHT	3	3	0.00501	**	0.00501	**	252.888		2	1.8	2.2
3000RCF	TW20	Size	SPIRAL	STRAIGHT	3	3	0.98	ns	0.98	ns	235.388		3	2.8	3.2
500RCF	KP188	Size	SPIRAL	STRAIGHT	3	3	0.762	ns	0.762	ns	257.588		1	0.8	1.2
500RCF	KP407	Size	SPIRAL	STRAIGHT	3	3	0.00657	**	0.00657	**	263.088		2	1.8	2.2
500RCF	TW20	Size	SPIRAL	STRAIGHT	3	3	0.47	ns	0.47	ns	246.488		3	2.8	3.2

Pairwise comparison_2 (t-test, Bonferroni adjust) Size															
Focal_Var	RIAC	.y.	group1	group2	n1	n2	p	p.signif	p.adj	p.adj.signif	y.position	groups	x	xmin	xmax
KP188	SPIRAL	Size	3000RCF	500RCF	3	3	0.591	ns	0.591	ns	257.588		1	0.8	1.2
KP407	SPIRAL	Size	3000RCF	500RCF	3	3	0.274	ns	0.274	ns	208.688		2	1.8	2.2
TW20	SPIRAL	Size	3000RCF	500RCF	3	3	0.0548	ns	0.0548	ns	232.788		3	2.8	3.2
KP188	STRAIGHT	Size	3000RCF	500RCF	3	3	0.0349	*	0.0349	*	270.588		1	0.8	1.2
KP407	STRAIGHT	Size	3000RCF	500RCF	3	3	0.518	ns	0.518	ns	263.088		2	1.8	2.2
TW20	STRAIGHT	Size	3000RCF	500RCF	3	3	0.203	ns	0.203	ns	246.488		3	2.8	3.2

Pairwise comparison_3 (t-test, Bonferroni adjust) Size														
RCF	RIAC	.y.	group1	group2	n1	n2	p	p.signif	p.adj	p.adj.signif	y.position	groups	xmin	xmax
3000RCF	SPIRA	Size	KP18	KP40	3	3	3.02	***	9.07E-	***	255.6		1	2

	L		8	7			E-04		04		88			
3000RCF	SPIRAL	Size	KP188	TW20	3	3	0.00637	**	0.0191	*	266.92		1	3
3000RCF	SPIRAL	Size	KP407	TW20	3	3	0.0155	*	0.0464	*	278.152		2	3
500RCF	SPIRAL	Size	KP188	KP407	3	3	0.00239	**	0.00717	**	257.588		1	2
500RCF	SPIRAL	Size	KP188	TW20	3	3	0.164	ns	0.491	ns	268.82		1	3
500RCF	SPIRAL	Size	KP407	TW20	3	3	0.0138	*	0.0415	*	280.052		2	3
3000RCF	STRAIGHT	Size	KP188	KP407	3	3	0.0455	*	0.137	ns	270.588		1	2
3000RCF	STRAIGHT	Size	KP188	TW20	3	3	0.00203	**	0.00608	**	281.82		1	3
3000RCF	STRAIGHT	Size	KP407	TW20	3	3	0.0367	*	0.11	ns	293.052		2	3
500RCF	STRAIGHT	Size	KP188	KP407	3	3	0.602	ns	1	ns	263.088		1	2
500RCF	STRAIGHT	Size	KP188	TW20	3	3	0.376	ns	1	ns	274.32		1	3
500RCF	STRAIGHT	Size	KP407	TW20	3	3	0.183	ns	0.548	ns	285.552		2	3

Pairwise comparison_4 (t-test, Bonferroni adjust) Size													
RIAC	.y.	group1	group2	n1	n2	p	p.signif	p.adj	p.adj.signif	y.position	groups	xmin	xmax
SPIRAL	Size	KP188	KP407	6	6	4.19E-07	****	1.26E-06	****	257.528		1	2
SPIRAL	Size	KP188	TW20	6	6	0.00166	**	0.00498	**	268.67		1	3
SPIRAL	Size	KP407	TW20	6	6	3.12E-04	***	9.37E-04	***	279.812		2	3
STRAIGHT	Size	KP188	KP407	6	6	0.294	ns	0.883	ns	270.528		1	2
STRAIGHT	Size	KP188	TW20	6	6	0.00269	**	0.00807	**	281.67		1	3
STRAIGHT	Size	KP407	TW20	6	6	0.0244	*	0.0733	ns	292.812		2	3
